# Effect of annealing temperature on the optoelectrical synapse behaviors of A-ZnO microtube

**DOI:** 10.1186/s11671-024-04060-2

**Published:** 2024-07-13

**Authors:** Yongman Pan, Qiang Wang, Anqing He, Yinzhou Yan, Xingzhong Cao, Peng Liu, Yijian Jiang

**Affiliations:** 1 School of Physics and Optoelectronic Engineering, Beijing University of Technology, Beijing, 100124 China; 2 College of New Materials and Chemical Engineering, Beijing Institute of Petrochemical Technology, Beijing, 102617 China; 3 College of Materials Science and Engineering, Beijing University of Technology, Beijing, 100124 China; 4 Institute of High Energy Physics, Chinese Academy of Sciences, Beijing, 100049 China

**Keywords:** Annealing, Optoelectrical synapse behaviors, Negative thermal quenching, “Learning-experience” behavior

## Abstract

Optoelectronic synapses with fast response, low power consumption, and memory function hold great potential in the future of artificial intelligence technologies. Herein, a strategy of annealing in oxygen ambient at different temperatures is presented to improve the optoelectronic synaptic behaviors of acceptor-rich ZnO (A-ZnO) microtubes. The basic synaptic functions of as-grown and annealed A-ZnO microtubes including excitatory postsynaptic current (EPSC), short-term memory (STM) to long-term memory (LTM) conversion, and paired-pulse facilitation (PPF), were successfully emulated. The results show that the annealing temperature of 600 °C yields high figures of merit compared to other annealed A-ZnO microtubes. The 4-fold and 20-fold enhancement dependent on the light pulse duration time and energy density have been achieved in the 600 °C annealed A-ZnO microtube, respectively. Furthermore, the device exhibited a PPF index of up to 238% and achieved four cycles of “learning-forgetting” process, proving its capability for optical information storage. The free exciton (FX) and donor–acceptor pair (DAP) concentrations significantly influenced the persistent photoconductivity (PPC) behavior of A-ZnO microtubes. Therefore, the LTM response can be controlled by the adjustment of numbers, powers, and interval time of the optical stimulation. This work outlines a strategy to improve the EPSC response through defect control, representing a step towards applications in the field of optoelectronic synaptic device.

**Supplementary Information:**

The online version contains supplementary material available at 10.1186/s11671-024-04060-2.

## Introduction

Optoelectronic synaptic devices integrating light-perception and signal-storage functions hold great potential in neuromorphic computing for visual information processing, as well as complex brain-like learning, memorizing, and reasoning [1–4]. Metal oxide materials are considered the first choice for the functional layer of optoelectronic synaptic devices owing to their compatibility with CMOS technology, among which ZnO is outstanding for its large exciton binding energy, rich surface defects, and excellent photo-absorption properties [5–7]. The optoelectronic synaptic performance of ZnO has been realized through invoking intrinsic defects. Recently, an Au/ZnO/Au structure was proposed to imitate habituation and sensitization behaviors under electrical and optical stimuli [8]. The response mechanism originated from the trapping states which is induced by oxygen vacancy (*V*_O_), and adsorption of oxygen at the interface of Au/ZnO. However, there is a lack of identification for the* V*_O_ defects. X-ray photoelectron spectroscopy (XPS), photoluminescence (PL) spectra, and positron annihilation lifetime spectrum (PALS) etc*.* are effective methods to validate the defects in ZnO [9–11]. Au/HfO_x_/ZnO/ITO heterostructure was presented to the optoelectronic synaptic device [12]. The device exhibited several basic synaptic functions such as EPSC, PPF, and learning-experience behaviors. The XPS spectra demonstrated that abundant oxygen defects present in the HfO_x_ layer served as trapping sites and modulated the internal dynamics of the photoelectrons. In addition, the annealing temperature of the defects strongly influenced the optoelectronic synaptic behaviors of ZnO. Al/ZnO/Al structure was designed to enhanced LTM properties [13]. The light-controlled synaptic device exhibited higher EPSC values, slower decay rates, and PPF behavior etc. The room temperature PL spectra demonstrated that the intensity of near band edge (NBE) emission in the ZnO annealed at 400 °C was increased, which contributed to the improved synaptic functionality. However, the NBE emission of ZnO includes abundant exciton emissions. The improved optoelectronic synaptic behaviors should further to be addressed. Therefore, the controllable defect in ZnO gives rise to a rich variety of information about carrier transport and optoelectronic synergetic modulation, enabling advanced optoelectronic devices with tunable properties.

Our previous work has demonstrated the stable shallow acceptor defect of zinc vacancy (*V*_Zn_) in ZnO (A-ZnO) microtubes/rods grown by optical vapour supersaturated precipitation (OVSP), with a binding energy of ~ 127 meV. Moreover, the *V*_Zn_ defects acted as an acceptor level, activating DAP recombination with intrinsic compensation donors of *V*_O_ for visible PL emission up to 773 K [14]. In addition, the negative thermal quenching effect at 200–240 K of temperature-dependent PL spectra was associated with thermal excitation of electrons in a deep level of 488 meV below the conduction band minimum (CBM) in A-ZnO microtube [15]. Due to the presence of the shallow acceptor defect in A-ZnO microtube, the electric performance is simultaneously influenced. The higher concentrations of *V*_Zn_ defects in A-ZnO microtubes reduced the electrical resistivity, resulting in significant resistive switching behavior with an enhanced on/off ratio of up to ∼10^3^ [16]. The regulation of *V*_Zn_ and *V*_O_ related defect concentrations achieved intrinsic color-tunable electroluminescence emission in a highly compensated ZnO microrod. The two and four potential channels of radiative recombination for free-electron-to-neutral-acceptor (FA) and DAP transition contributed to the EL emission energy [17]. Inspired by the reliable defect regulation of A-ZnO microtube, the effect of annealing temperature on the optoelectrical synapse behaviors of A-ZnO microtube was developed.

In this work, the high temperature annealing in oxygen atmosphere method was utilized to improve the optoelectrical synapse behaviors of A-ZnO microtube. The basic synaptic functions including the EPSC, STM transition to LTM, PPF, and learning-experience behavior of A-ZnO microtube were successfully demonstrated. The STM transition to LTM has also been demonstrated by increasing the light pulse of duration time, number, energy density, and interval time. Benefiting from the shallow donor defect and the higher concentrations of shallow acceptor level, the PPC behavior of A-ZnO microtubes can be significantly improved. The presence of defect level was confirmed by the PL, PALS, and XPS spectra. Therefore, the defect-control optoelectrical response mechanism was revealed. Such a study opens up new opportunities to design novel wide-bandgap semiconductor optoelectrical synapse devices with a simple structure for high-efficiency synaptic plasticity.

## Experimental methods

### Preparation of A-ZnO microtube

The as-grown A-ZnO microtubes were grown by the OVSP method, detailed preparation conditions can be found in our previous work [14]. The as-grown A-ZnO microtubes were then annealed in a forming O_2_ environment with a flow rate of 10 mL/min. The annealed temperature was set in the range of 400–800 °C for 1 h. The working pressure was set at 0.3 MPa. The standard undoped n-ZnO single crystal bulk with an orientation of < 0001 > and a Zn-ending-surface was used as control, of which the size was 5 × 5 × 0.5 mm^3^ (purchased from Hefei Kejing Material Technology Co., Ltd).

### Apparatus and characterization

The microstructure of A-ZnO microtube was examined by a high-resolution transmission electron microscopy (HRTEM) and selected area diffraction patterns (SAED). The cross-sectional view of the A-ZnO microtube was obtained by a scanning electron microscopy (SEM). The coordination environment of A-ZnO microtubes was analyzed by X-ray absorption fine structure (XAFS) measurements on 1W2B beam line at Beijing Synchrotron Radiation Facility (BSRF). The ultraviolet–visible (UV–vis) absorption spectrum was recorded by an UV–vis spectrophotometer, with BaSO_4_ powder as the reference. The Raman spectra of A-ZnO microtube were acquired by a SmartRaman confocal-micro-Raman system (developed by Institute of Semiconductors, Chinese Academy of Science) equipped with a Horiba LabRAM iHR550 spectrometer, under the backscattering geometry with a 2400 lines mm^−1^ grating and a CCD detector. For the defect-dependent longitudinal optical (LA) phonon Raman modes analysis, a 633-nm He–Ne linear polarization laser (Thorlabs HNL210) beam was set to be perpendicular to the *c*-axis of the A-ZnO microtube by a 20 × /*NA*0.40 objective (Olympus MPLN20 ×) with a spot size of ~ 2 μm. The room and temperature-dependent PL spectra of A-ZnO microtubes were captured by the same spectrometer with a 600 lines/mm grating. The excitation wavelength was 325 nm from a CW He-Cd laser with a 5 × /*NA*0.13 objective (Thorlabs LMU-5 × -NUV).

The concentrations of defects in A-ZnO microtubes were characterized by PALS measurements facility at Institute of High Energy Physics (IHEP). The radioactive isotopes of ^22^Na was used as the positron source. The time resolution of the spectrometer is about 210 ps. The PALS data can be analyzed with deconvolution using specialized program LT9.0. The surface chemical states of A-ZnO microtubes were obtained from XPS spectra. The binding energies were calibrated using C1*s* peak at 284.8 eV to compensate the surface charges.

The optoelectrical synapse behavior of A-ZnO microtube was measured with a house-made system, including a semiconductor parameter analyzer (Keithley 2636B), an unfocused pulse laser with a wavelength of 355 nm, a pulse width of 5 ns, a frequency of 30 Hz, and TTL-controlled optical shutters. An optical power meter (Newport Model 843-R) was employed to obtain the energy density of optical pulses. An individual A-ZnO microtube was placed onto a clean glass substrate by a high precision tweezer. The graphite electrodes were then deposited onto both ends of the A-ZnO microtube by a needle tubing. The distance of the A-ZnO microtube between two electrodes was ~ 0.9 mm.

## Results and discussion

### Schematic diagram of A-ZnO microtube optoelectrical synapse

**Fig. 1:**
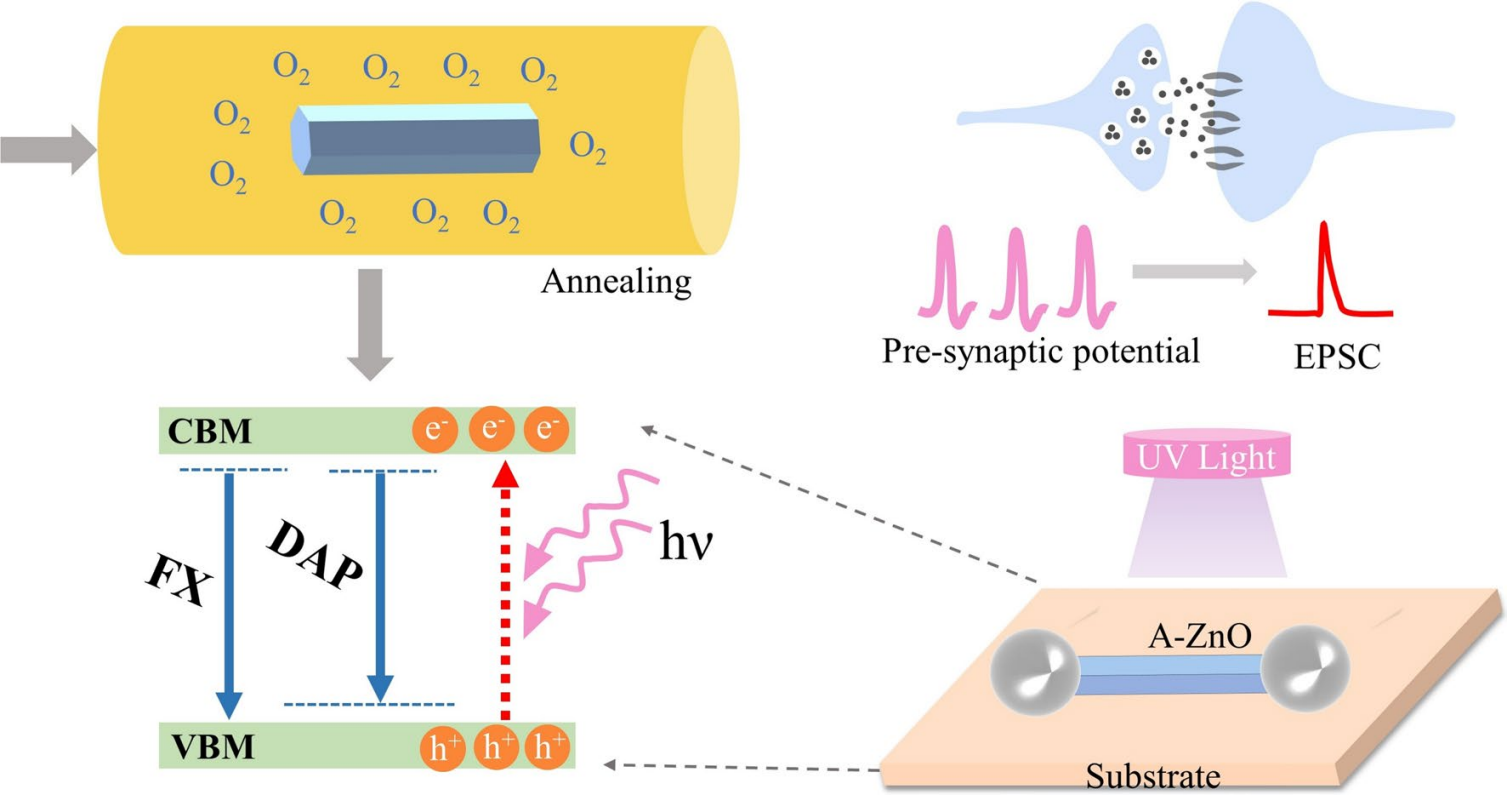
Schematic of A-ZnO microtube optoelectrical synapse device

Figure 1 shows the concept of achieving reliable optoelectrical synapse behaviors of A-ZnO microtubes that were annealed in the oxygen atmosphere. The annealing process influences the concentration of carriers and behavior of defects in A-ZnO microtube such as FX and DAP emissions etc*.*, which contribute to reveal the mechanism of optoelectrical synapse behaviors. The UV light pulse mimicks the presynaptic potential, the photocurrent of A-ZnO microtube represents EPSC response as the postsynaptic signal. The variation of EPSC response is regarded as the synaptic weight, indicating how optical information is transferred from presynaptic to post-synaptic neurons.

### Morphological and structural characterization of as-grown and annealed A-ZnO microtubes

**Fig. 2:**
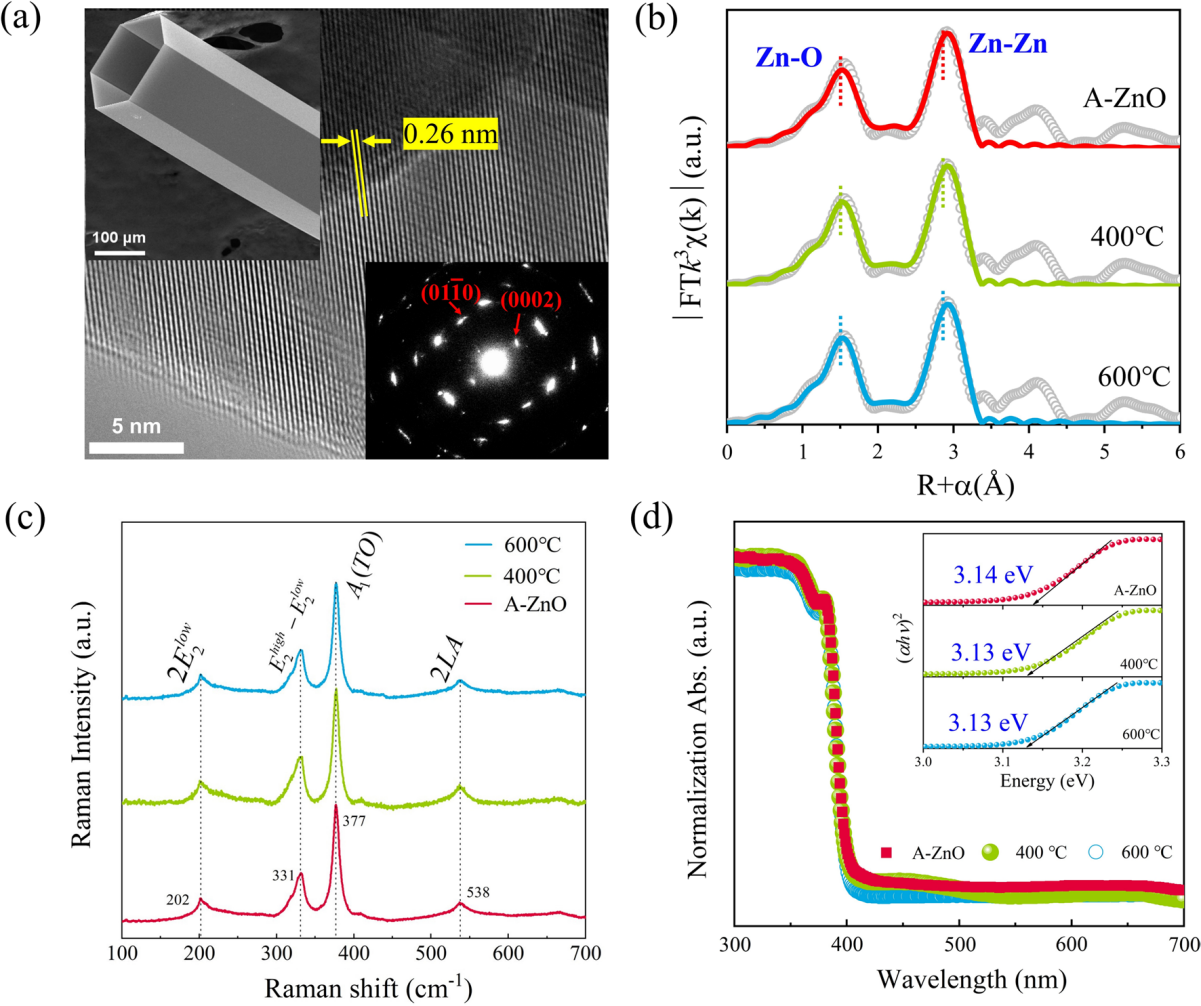
Characterization of as-grown and annealed A-ZnO microtubes. **a** The as-grown A-ZnO microtube of TEM image, left top inset: SEM image, right down inset: SAED pattern. The as-grown and annealed A-ZnO microtubes of **b** Fourier transform *k*^3^-weighted XAFS spectra, **c** Raman spectra, and **d** the UV–vis spectra. Inset: Tauc plot extracted from the absorption spectra

Figure 2a shows a HRTEM image taken from the edge of as-grown A-ZnO microtube. The lattice spacing of 0.26 nm matches the (0002) plane of the A-ZnO microtube [18]. The corresponding SAED image of A-ZnO microtube is shown in the lower right inset of Fig. 2a. The presence of sharp diffraction spots indicates the high crystal quality of the A-ZnO microtube. The shape of the A-ZnO microtube, possessing a hexagonal architecture and well-faceted sidewalls, is shown in the upper left inset of Fig. 2a. The Fourier-transformed *k*^3^-weighted XAFS spectra of as-grown and annealed A-ZnO microtubes are shown in Fig. 2b, and the corresponding curve-fitting results are summarized in Table S1. It is noted that the coordination number (N) of Zn–O (A-ZnO: N = 3.9, 400 °C: N = 3.5, 600 °C: N = 3.5) and Zn–Zn (A-ZnO: N = 11.8, 400 °C: N = 11.7, 600 °C: N = 11.6) slightly decreased with increasing annealing temperature. The annealing temperature has little influence on the bond length of Zn–O (1.96 Å) [19]. The Zn–Zn bond is elongated from 3.22 Å for A-ZnO microtube to 3.23 Å for 600 °C annealed A-ZnO microtube [20]. These results suggest that there is slight disorder in the second shell around the Zn atoms of the A-ZnO microtube.

The Raman spectra of as-grown and annealed A-ZnO microtubes are shown in Fig. 2c. The Raman shifts located at 202 cm^−1^, 331 cm^−1^, 377 cm^−1^, and 538 cm^−1^ correspond to the 2*E*_low,2_, *E*_high,2_–*E*_low,2_, A_1_(TO), and 2LA modes, respectively [21]. The 2LA modes suggest the presence of *V*_Zn_ related defects in A-ZnO microtube [22]. Figure 2d exhibits the absorbance edge at ~ 390 nm. The calculated values from Tauc plot in the right top inset of Fig. 2d showed that the optical bandgap (*E*_g_) of as-grown and annealed A-ZnO microtubes to be 3.14 eV and 3.13 eV, respectively. This indicated that more photogenerated electron–hole pairs will be generated with narrowing *E*_g_ for the annealed A-ZnO microtubes [23], which benefits the realization of optoelectrical synaptic behaviors.

### Synaptic plasticity of as-grown and annealed A-ZnO microtubes

**Fig. 3:**
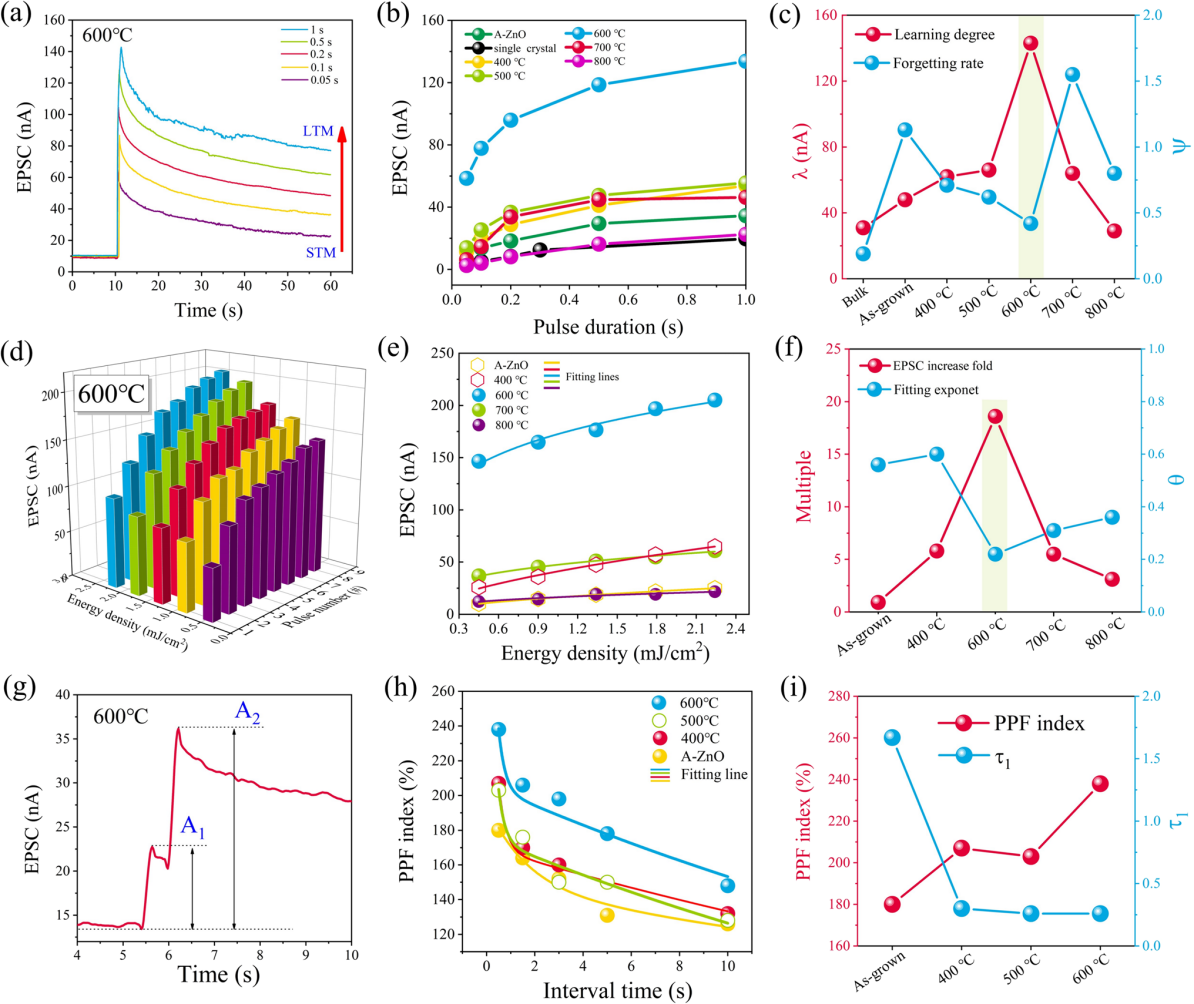
The synapse plasticity of as-grown and annealed A-ZnO microtubes. Dependence of light pulse parameters on the EPSC response: **a**–**c** duration time; **d**–**f** energy density; and **g**–**i** interval time

Figure 3a and Fig. S1 demonstrate the STM transition to LTM process under different light pulse duration time for the as-grown and annealed A-ZnO microtubes [24], respectively, with ZnO single crystal bulk as control. Figure 3b and Fig. S2 show that the EPSC value of annealed A-ZnO microtubes gradually increased with the temperature increase from 400 °C to 600 °C. However, raising the annealing temperature above 700 °C results in the appearance of weakening the EPSC response. The comparison relative EPSC values of the as-grown and annealed A-ZnO microtubes are listed in Table S2–S3. The results show that the annealing temperature of 600 °C with high figures of merit as compared to other annealed A-ZnO microtubes. The biological forgetting law can be fitted by the Wickelgren’s power-law model [25]:

1 $$I = \lambda \times {(1 + }\beta \times t{)}^{ - \psi }$$

where *I* is the memory level, *t* is the decay time, *λ* is the learning degree, *β* is a scale parameter, and ψ is the forgetting rate. The fitted learning degree and forgetting rate of as-grown and annealed A-ZnO microtubes under light pulse duration time of 1s, as displayed in Fig. 3c. It was observed that the 600 °C annealed A-ZnO microtube exhibited a remarkable learning degree and a slowly forgetting rate.

Figure 3d and Fig. S3 exhibit the STM can be converted into LTM by increasing the number and energy density of light pulses in the A-ZnO microtubes as well. In comparison with the as-grown A-ZnO microtube, the EPSC value of annealed samples gradually increased as the temperature increased from 400 °C to 600 °C while decreased when the temperature increased from 700 °C to 800 °C, as shown in Fig. 3e and Fig. S4.

The corresponding enhancement of EPSC are summarized in Table S1. The 20-fold enhancement of EPSC response was observed for the 600 °C annealed A-ZnO microtube in comparison with that of the as-grown sample, as shown in Fig. 3f. In addition, the dependence of EPSC on light pulse energy density can be fitted by a power law [26]:

2 $$I = {\text{A}}P^{\theta }$$

where *I*, A, *P*, and *θ* are EPSC, a constant, energy density, and the index of power law, respectively. The value of *θ* is fitting to be 0.56, 0.60, 0.22, 0.31, and 0.36 for the as-grown and annealed A-ZnO microtube, respectively, as shown in Fig. 3f. All the values of *θ* < 1, implying that the complex process of the photocarriers in the A-ZnO microtube involves both trap/defect states and photogenerated carrier interactions [27, 28].

PPF is an essential learning rule of biological synapse, where postsynaptic stimulation can evoke a reinforced EPSC that depends on the interval time (Δt) between consecutive pairs of light pulses [24]. The PPF value is defined by the ratio of A_2_ and A_1_, where A_1_ and A_2_ are the peak amplitudes of current from the first and second light pulse, respectively. Figure 3g and Fig. S5-S8 show the PPF behavior of the as-grown and annealed A-ZnO microtube. The maximum PPF index of 238% was achieved in the 600 °C annealed A-ZnO microtube, while the PPF index of the as-grown, 400 °C annealed and 500 °C annealed A-ZnO microtube were 180%, 207%, and 203%, respectively, as shown in Fig. 3h and listed in Table S5-S6. A short (larger) Δt resulted in a larger (smaller) EPSC under stimulation of the second spike [29]. The PPF index of 238% is better than that of other reported ZnO-based optoelectrical synapse device, as listed in Table S7. The PPF relaxation characteristics dependence on Δt for the as-grown and annealed A-ZnO microtubes are shown in Fig. 3i. The relaxation times can be fitted by a double-exponential function as follows [30]:

3 $${\text{PPF }} = {\text{ }}1{\text{ }} + {\text{ }}C_{1} \times \exp \left( {\frac{{ - \Delta t}}{{\tau _{1} }}} \right){\text{ }} + {\text{ }}C_{2} \times \exp \left( {\frac{{ - \Delta t}}{{\tau _{2} }}} \right)$$

where Δt is the pulse interval time, C_1_ and C_2_ are the initial facilitation magnitudes of the respective phases, and *τ*_1_ and *τ*_2_ are the characteristic relaxation time of the rapid and slow phases, respectively. The *τ*_1_ and *τ*_2_ values are 0.26 s and 33.79 s for the annealed 600 °C A-ZnO microtube, respectively, which are compatible with the values in biological synapses [31]. Therefore, the annealing temperature improved the PPF behavior of the as-grown A-ZnO microtube.

### Dependence of annealing temperature on the optical properties of A-ZnO microtube

**Fig. 4:**
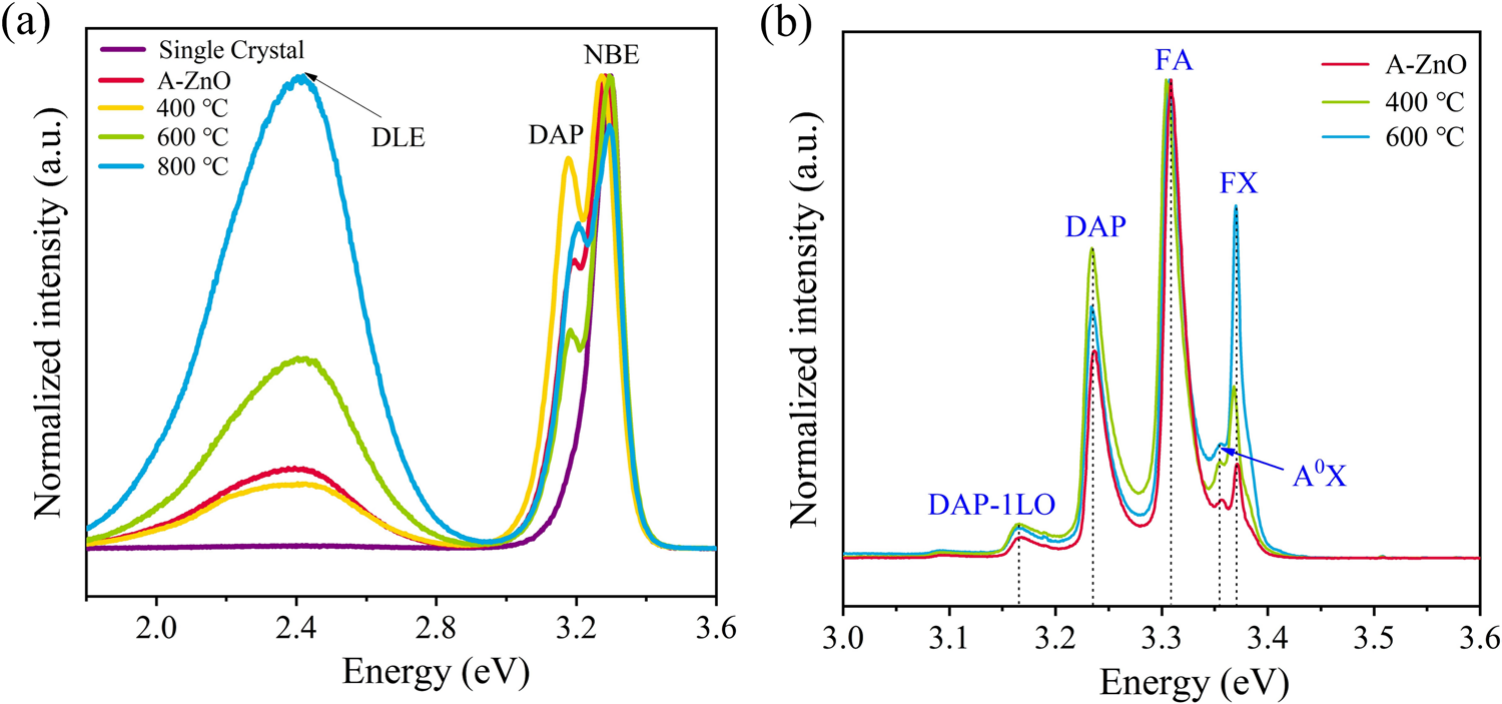
Comparison of PL spectra for the as-grown and annealed A-ZnO microtube at: **a** 300 K, and **b** 80 K, respectively

Figure 4a shows PL spectra at 300 K of the as-grown and annealed A-ZnO microtubes, with ZnO single crystal bulk used as the control. The PL emission peaks located at 3.29 eV, 3.17 eV, and 2.45 eV were attributed to NBE, DAP, and deep-defect level (DLE) emission, respectively. The presence of DAP emissions indicated the existence of *V*_Zn_ defects in the A-ZnO microtube, which was absent in the ZnO single crystal bulk. The high recombination rate of the DLE suppressed the NBE emission in the 800 °C annealed A-ZnO microtube, suggesting low optical quality. The high-temperature annealing provides enough ionization energy and increases the concentration of *V*_O_ defects, strengthening the DLE emission [32]. Therefore, the EPSC response of the 800 °C annealed sample was lower than that of the as-grown A-ZnO microtube, as shown in Fig. 3b. In addition, the Gaussian fitting of the DLE emission of the as-grown and annealed A-ZnO microtubes indicated that the defect concentrations increased with the annealing temperature [33], as shown in Fig. S9 and Table S8.

For further insight into the dependence of annealing temperature on NBE emission, PL spectra at 80 K of the as-grown and annealed A-ZnO microtubes are shown in Fig. 4b. The PL emission peaks located at 3.37 eV, 3.35 eV, 3.31 eV, 3.24 eV, and 3.17 eV were associated with the FX, neutral acceptor-bound excitons (A^0^X), FA, DAP, and longitudinal optical (LO) phonon replicas of DAP emission, respectively [34]. Moreover, the intensities of these emissions increased significantly with the annealing temperature of 400 °C and 600 °C. The relative intensity ratios of FX and DAP with respect to the dominant FA emission for the 600 °C annealed sample were enhanced by 3.7-fold and 1.5-fold, respectively, compared with the as-grown A-ZnO microtube. The FX emission is evidence of a higher crystalline quality of the annealed A-ZnO microtube [35]. In addition, the binding energy (*E*_A_) of the *V*_Zn_ defect is given by [36]:

4 $$E_{{\text{A}}} = E_{{\text{g}}} - E_{{{\text{FA}}}} + \frac{{k_{{\text{B}}} T}}{2}$$

where *E*g is the ZnO band gap energy at low temperature (*E*g = 3.437 eV), *E*_FA_ is the FA transition energy, *k*_B_ is Boltzmann’s constant, and *T* is the temperature, respectively. The corresponding *E*_A_ value showed that the shallow acceptor level of the *V*_Zn_ defect is located 130 meV above the valence band maximum (VBM). Therefore, the FX and DAP emissions of the 600 °C annealed A-ZnO microtube were both improved.

**Fig. 5:**
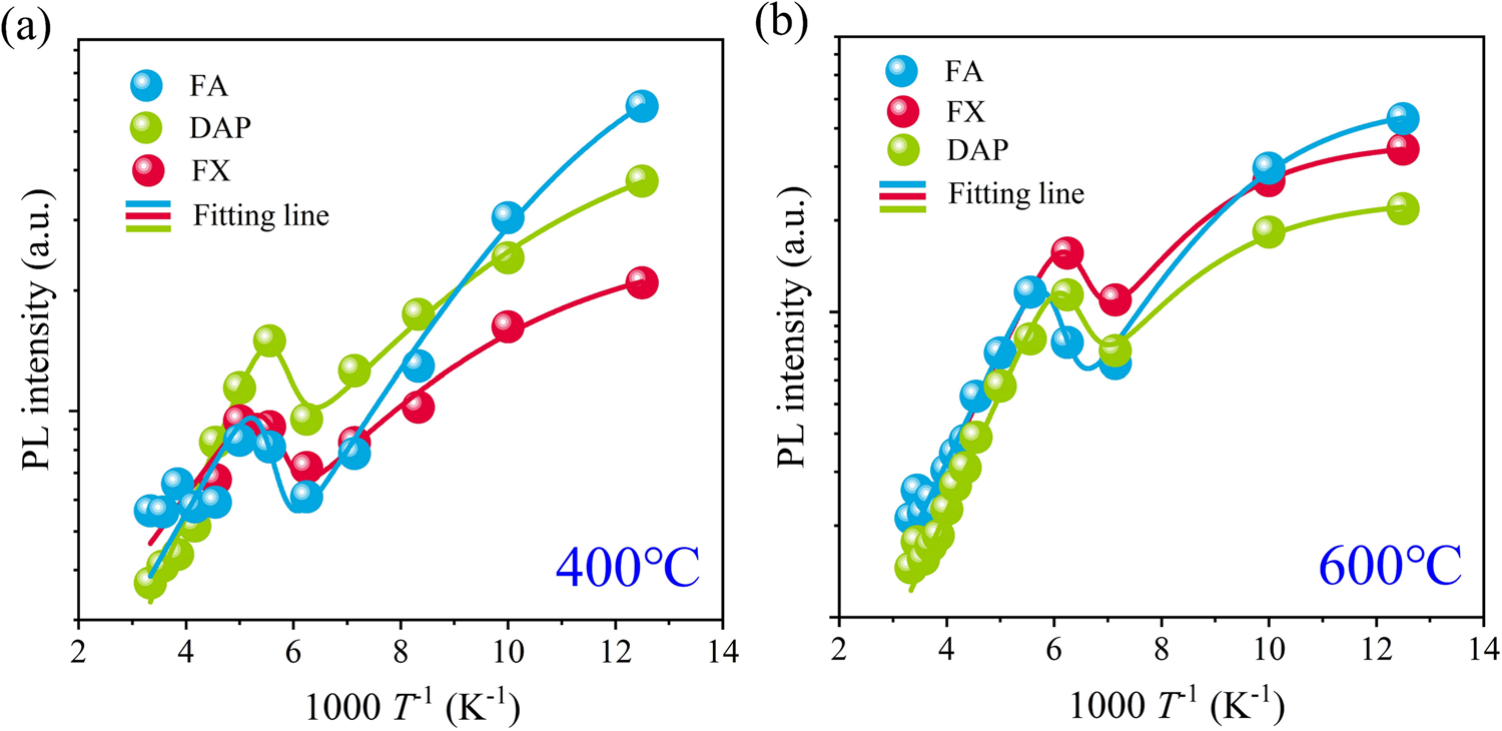
Dependence of annealing temperature on the peak intensity of A-ZnO microtube: **a** 400 °C and **b** 600 °C

Temperature-dependent PL spectra were conducted in order to understand how annealing induces the enhancement of excitonic emission in the as-grown A-ZnO microtube. The intensities of FA, DAP, and FX emission decreased with the PL temperature in the range of 80–160 K and 260 –300 K, while it increased in the middle range of 180–240 K for the 400 °C annealed A-ZnO microtube, as shown in Fig. S10(a-c). The anomalous PL emission behavior was attributed to the negative thermal quenching (NTQ) effect. At 80 K of PL spectra, the maximum PL emission intensity was for FA, DAP, and FX, in good agreement with the as-grown A-ZnO microtube as reported previously [15]. However, the order switched to FA, FX, and DAP emissions for the 600 °C annealed A-ZnO microtube, as shown in Fig.S10(d-f). Furthermore, the NTQ effect was observed in the temperature ranges of 80–160 K and 250–300 K. Therefore, the 600 °C annealed A-ZnO microtube exhibited a stronger NTQ effect. The NTQ behavior can be explained by using a multilevel model expressed as follows [37]:

5 $$I(T) = I(0)\frac{{1 + \sum\limits_{q = 1}^{w} {D_{q} \exp ( - E_{q}^{\prime} /{\text{k}}_{{\text{B}}} T)} }}{{1 + \sum\limits_{j = 1}^{m} {C_{j} \exp ( - E_{j} /{\text{k}}_{{\text{B}}} T)} }}$$

where *I(T)* is the PL intensity at measured temperature *T* (K), *I(0)* is the PL intensity at 0 K. *Dq* and *Cj* are fitting parameters. *k*_B_ is the Boltzmann constant. The activation energy $E_{q}^{\prime}$ describes the thermal quenching of the PL intensity, and the activation energy *Ej* represents the non-radiative channel. *W* and *m* indicate the number of intermediate states and non-radiative recombination channels, respectively. The temperature dependence of the PL intensity was well fitted with one (*w* = 1) intermediate state and two (*m* = 2) non-radiative recombination channels for the 400 °C and 600 °C annealed A-ZnO microtube, as shown in Fig. 5a–b, respectively. The obtained activation energies are listed in Table 1.

**Table 1:** Fitting activation energies for the annealed A-ZnO microtube

Exciton emission	$E_{q}^{\prime}$ (400 °C/600 °C)	*E*_1_ (400 °C/600 °C)	*E*_2_(400 °C/600 °C)
FA → NBE	475/426	522/499	45/62
FX → NBE	420/366	456/439	35/64
DAP	399/358	463/435	34/59

**Fig. 6:**
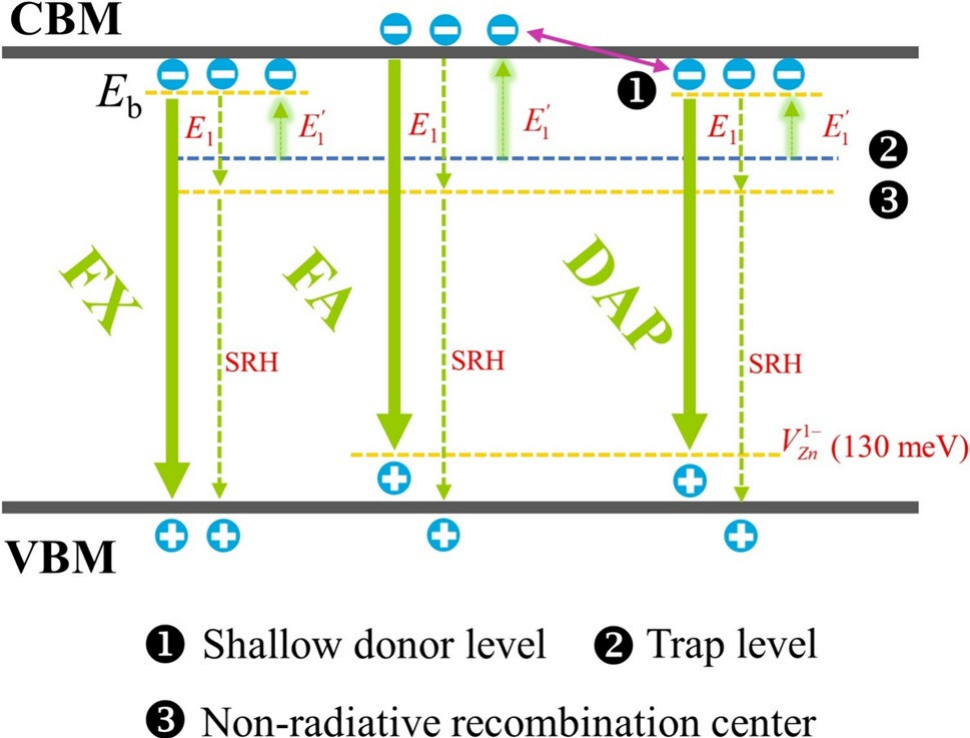
Schematic diagram of the NTQ effect in annealed A-ZnO microtube

From the fitting results, the $E_{1}^{\prime}$ differences of DAP to FA and FX to FA was 58 meV and 77 meV for the 400 °C annealed A-ZnO microtube, respectively, which was close to the FX binding energy of 60 meV (*E*_b_) in ZnO. Therefore, a shallow trap level of 475 meV below the conduction band minimum (CBM) is responsible for the intermediate state, which is a characteristic of the NTQ effect. Similarly, the lower shallow trap level of the 600 °C annealed A-ZnO microtube was 426 meV. The* E*_1_ value of the 400 °C and 600 °C annealed A-ZnO microtube was 522 meV and 456 meV, indicating that the existence of a non-radiative recombination channel based on the Shockley–Read–Hall (SRH) recombination. *E*_2_ is too shallow to represent an effective non-radiative recombination center according to previous reports [11]. The charged defect of *V*_O_ possessed transition levels (1 + /0) and (2 + /1 +) in the range of 2.7–2.9 eV above the VBM, respectively [38]. Consequently, the activation energies of $E_{1}^{\prime}$ and *E*_1_ are related to the defect level of *V*_O_. With increasing temperature, localized carriers in the shallow trap level were activated, then transferred to the CBM, resulting in enhanced FX and DAP emissions of the annealed A-ZnO microtube, as shown in Fig. 6.

**Fig. 7:**
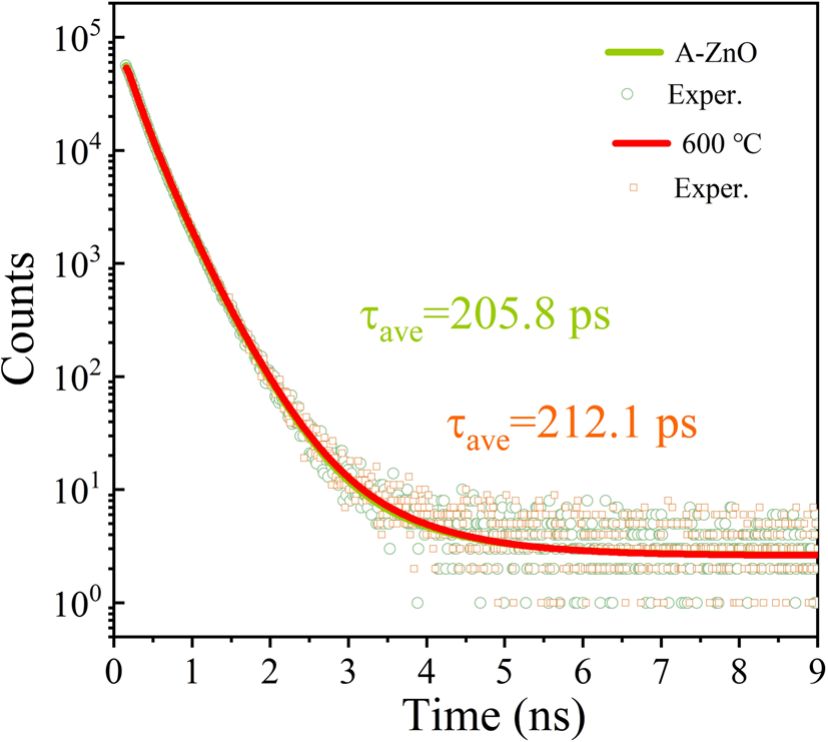
PALS spectra of as-grown and 600 °C annealed A-ZnO microtube

Since positrons are sensitive to neutral or negatively charged defects, the PALS spectra are employed to characterize the *V*_Zn_ defect of the A-ZnO microtube, as shown in Fig. 7. The average lifetime (τ_ave_) of positrons can be calculated as follows:

6 $$\tau_{ave} = \tau_{1} I_{1} + \tau_{2} I_{2} + \tau_{3} I_{3}$$

where the short-lifetime *τ*_1_ is attributed to free annihilations of positrons and self-annihilations of para-positronium (p-Ps), the intermediate-lifetime *τ*_2_ originates from the annihilation of trapped positrons at the defects, the longest-lifetime *τ*_3_ represents the pick-off annihilations of the ortho-positronium (o-Ps). *I*_1_, *I*_2_, and *I*_3_ are the probabilities of positrons annihilated in the specimen [39]. The fitting results are listed in Table S9. The τ_ave_ value of the 600 °C annealed sample is ~ 212 ps, which shows a prolongation of 3.1% in comparison with the as-grown A-ZnO microtube, validating the high concentration of DAP from the *V*_Zn_ defect. Based on the previous studies, the positron lifetime in ZnO is measured to be ~ 170 ps and that for positrons trapped in negatively charged *V*_Zn_ is supposed to be ~ 215 ps [40]. Therefore, it is confidently considered that the presence of *V*_Zn_ defect with 1 + positive charges is in good agreement with the PL spectra.

### XPS of as-grown and annealed A-ZnO microtubes

**Fig. 8:**
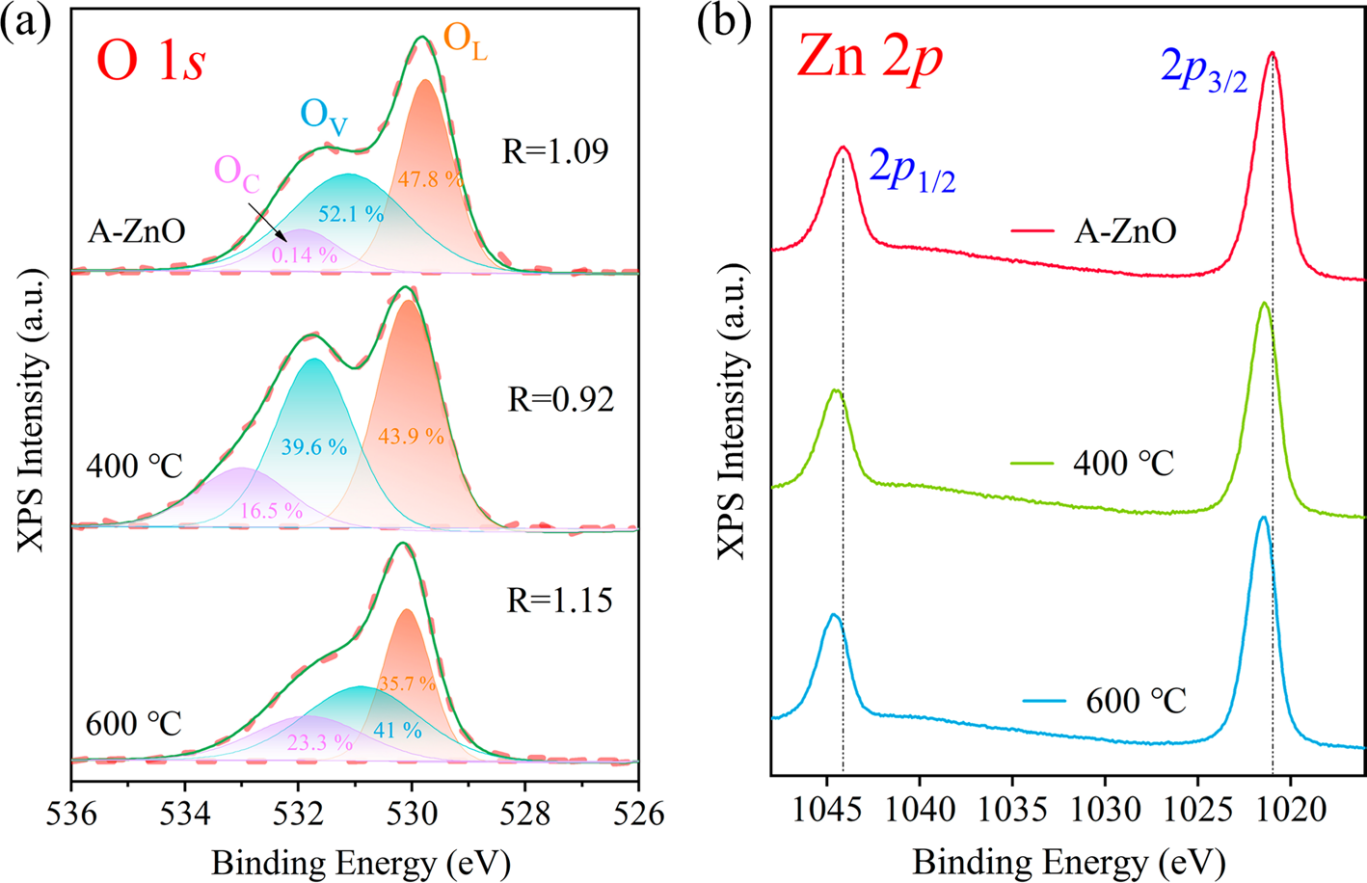
XPS spectra for the as-grown and annealed A-ZnO microtube of **a** O 1s, and **b** Zn 2p

The oxidation states and chemical compositions of the as-grown and annealed A-ZnO microtubes were investigated quantitatively by XPS fine spectra. Figure 8a shows that the O 1*s* peak could be fitted with three components by Gaussian–Lorentzian, corresponding to the chemisorbed oxygen species (O_*C*_), oxygen vacancies (O_*V*_), and lattice oxygen (O_*L*_), respectively. *R* was estimated by the peak area ratio of O_*V*_ to O_*L*_ to determine the concentration of *V*_O_. The as-grown and 400 °C and 600 °C annealed A-ZnO microtubes exhibited *R* values of 1.09, 0.92, and 1.15, respectively. Figure 8b exhibits the difference between Zn 2*p*_1/2_ and Zn 2*p*_3/2_ peak positions was found to be ~ 23 eV, which indicated the presence of divalent zinc ions (Zn^2+^) [41]. The obtained binding energy of O 1*s* and Zn 2*p* from the as-grown and annealed A-ZnO microtubes, are listed in Table S10, respectively. The Zn 2*p* binding energy of annealed A-ZnO microtubes clearly shifted to higher energy, implying a decreased number of outer-shell electrons [42]. The capability of annealing temperature to regulate the defect concentration was further supported.

### The working mechanism of as-grown and annealed A-ZnO microtubes optoelectrical synapse

Based on the above results, the synaptic plasticity is significantly affected by the annealing temperature for the as-grown A-ZnO microtubes. Therefore, it is crucial to establish a physical mechanism of photocarriers excitation and relaxation processes during the EPSC response.

**Fig. 9:**
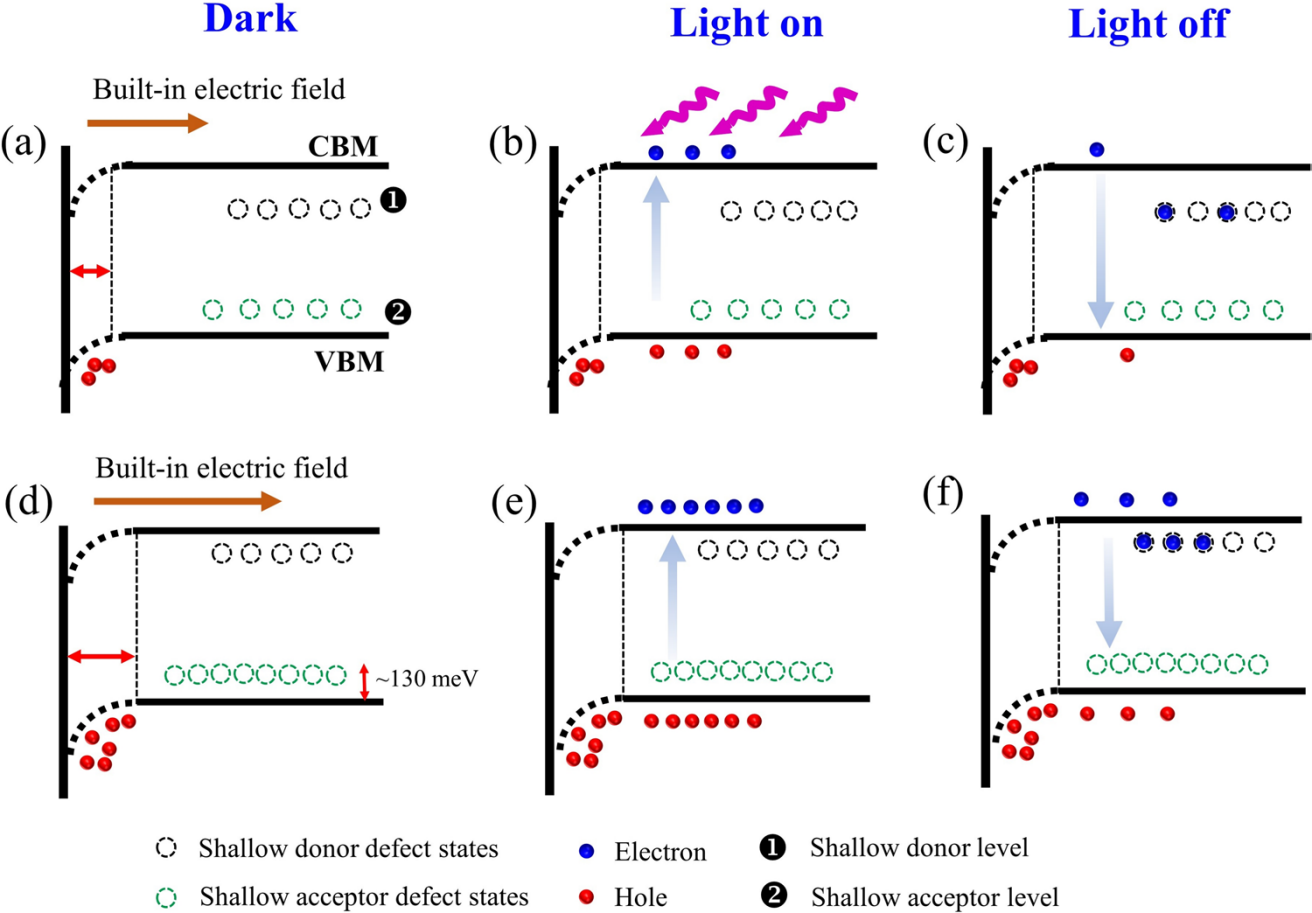
Band diagrams of **a**–**c** as-grown and **d**–**f** 600 °C annealed A-ZnO microtube under dark, light on, and light off condition, respectively

In the initial dark state, a built-in electric field is formed in the as-grown and 600 °C annealed A-ZnO microtubes due to the presence of a shallow acceptor level of *V*_Zn_ defect [14]. Meanwhile, the shallow donor defect level adjacent to the CBM as electron trap states were formed, as shown in Fig. 9a, d. When switching on light pulse in the as-grown A-ZnO microtube, photo-generated electron–hole pairs can be separated quickly by the built-in electric field, resulting in an increased EPSC as shown in Fig. 9b [43]. As for the 600 °C annealed A-ZnO microtube, the higher concentration of *V*_Zn_ defects contributed to a stronger built-in electric field, achieving an excellent light sensitivity, as shown in Fig. 9e. Thus, with the increase in the duration time, energy density, or number of light pulses, the EPSC response significantly increased. After turning off the light pulse, the photocarriers gradually transitioned back to the VBM, as shown in Fig. 9c, f. Since the shallow donor state trap electrons, extending the lifetime of electrons, thus leading to PPC behaviors [44, 45]. The recombination of *V*_O_ with non-lattice oxygen ions further contributed to the conductance decay [46]. Therefore, the memory level of EPSC for the as-grown A-ZnO microtube exhibited relatively lower than 600 °C annealed A-ZnO microtube, attributing this to fewer photocarriers participated in the conduction.

### Learning experience behavior of annealed A-ZnO microtube

**Fig. 10:**
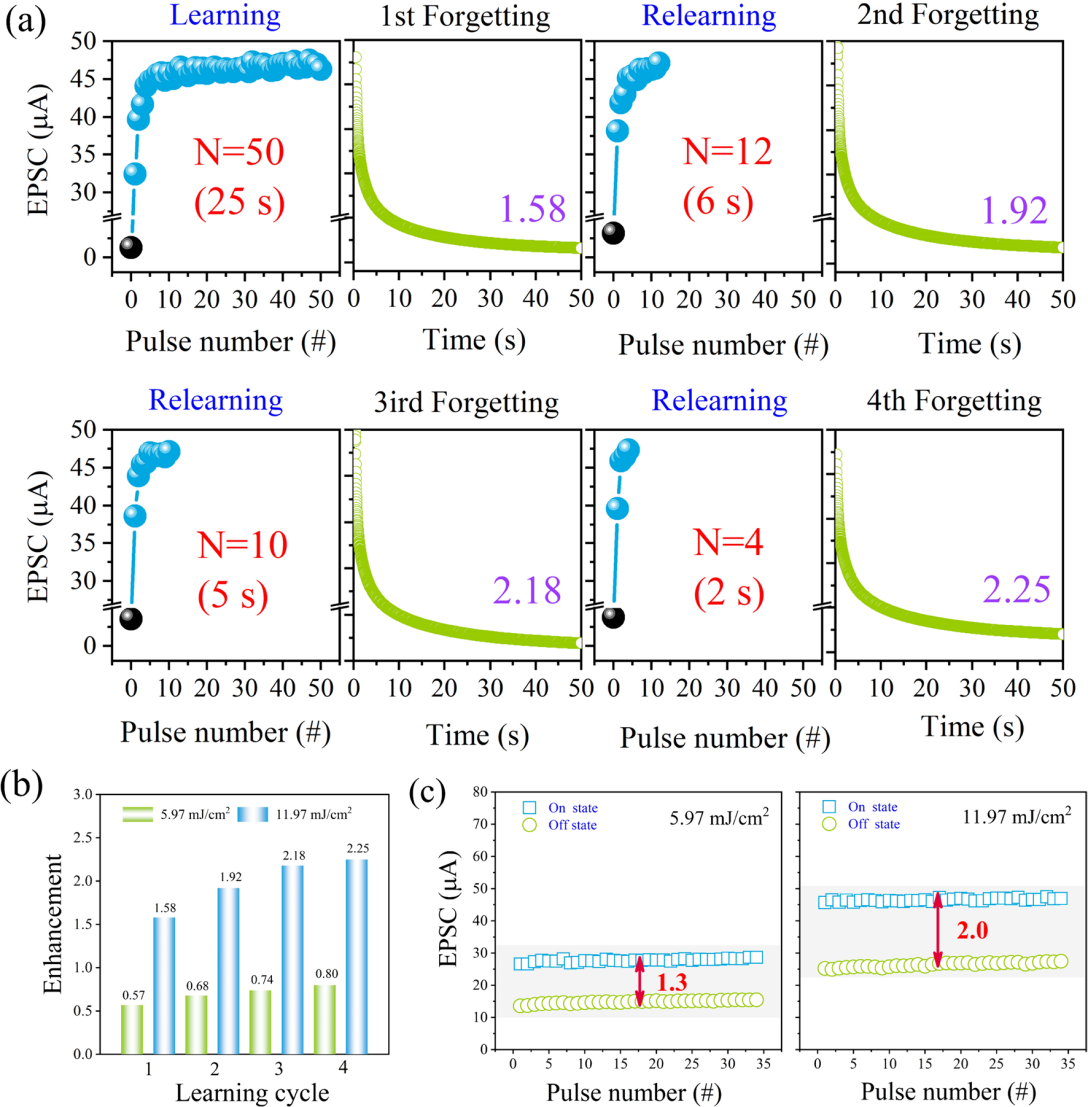
Learning-experience behavior of annealed 600 °C A-ZnO microtube. **a** Four cycles of “learning-forgetting” training. The black and blue solid ball represents the initial dark current and EPSC response, respectively. **b** Compared the enhancement EPSC with various energy densities. **c** After training, extracted the maximum and minimum values of EPSC from the 30 light pulses, respectively

Taking advantage of the remarkable EPSC response of the 600 °C annealed A-ZnO microtube, the learning experience of biological synapse was mimicked. The learning and memory process was stimulated by four light sequence trainings, each consisting of 50 light pulses at 355 nm, with each pulse lasting 0.5 s and an interval time of 0.05 s between pulses, and at a read voltage of 10 mV, followed by a spontaneous drop of 50 s (removing the light pulse). The light-on state represents the process of learning or relearning, and the light-off state corresponds to the forgetting process [46]. Fig. S11 and Fig. 10a depict the memory behavior of the 600 °C annealed A-ZnO microtube after applying identical light pulse with different energy densities of 5.97 mJ/cm^2^ and 11.97 mJ/cm^2^, resulting in a similar learning experience. During each of learning and forgetting process, a rapid increase of EPSC can be observed, followed by a slow decay, demonstrating a phenomenon similar to the memory and forgetting behavior of a biological synapse. Furthermore, the number of required light pulse decreased to 50, 12, 10, and 4 after repeating the learning or relearning operations. Therefore, the relearning previously acquired information can greatly boost memory enhancement, as shown in Fig. 10b. In addition, the maximum and minimum values of EPSC from the first cycle, dependent on various energy densities, are plotted in Fig. 10c The multi-conductance states demonstrated by the present device will be beneficial for realizing effective neuromorphic computing in the future [48].

## Conclusions

In summary, the optoelectrical synaptic plasticity of as-grown A-ZnO microtubes dependence on annealing in oxygen atmosphere at the temperature of 400–800 °C was investigated. Furthermore, the PPF and learning experience behavior of biological synapses were successfully achieved. The 600 °C annealed A-ZnO microtube exhibited superior capability to memorize light information, which showed a fourfold and 20-fold enhancement in terms of light pulse duration time and energy density dependence in comparison with that of the as-grown A-ZnO microtube. The value of the PPF index was increased to 238% for the 600 °C annealed A-ZnO microtube. The optoelectrical synaptic mechanism of EPSC enhancement is attributed to the high concentration of shallow acceptor defects. Meanwhile, the presence of shallow donor defect states acted as a trap level extending the lifetime of electrons, therefore increasing the memory level. The defect level was verified using temperature-dependent PL, PALS, and XPS spectra. The present work shows the applications of A-ZnO microtube for high-efficiency optoelectronic synapse devices in future.

### Supplementary Information

Supplementary File 1 (DOCX 20630 KB)

## Data Availability

Data is provided within the manuscript and supplementary information files.
