# Supplementary material for: Effect of annealing temperature on the optoelectrical synapse behaviors of A-ZnO microtube
Source: Discov Nano. 2024 Jul 13;19(1):116. doi: 10.1186/s11671-024-04060-2 (PMC11246399; doi:10.1186/s11671-024-04060-2)
Supplement: Supplementary file 1 — (DOCX 20630 KB) [file 11671_2024_4060_MOESM1_ESM.docx]

**Supporting Information**

**Effect of annealing temperature on the optoelectrical synapse behaviors of A-ZnO microtube**

Yongman Pan^1^, Qiang Wang^2^, Anqing He^3^, Yinzhou Yan^1^, Xingzhong Cao^4^, Peng Liu^4^, Yijian Jiang^1*^

*^1^ School of Physics and Optoelectronic Engineering, Beijing University of Technology, Beijing 100124, China*

*^2^ College of New Materials and Chemical Engineering, Beijing Institute of Petrochemical Technology, Beijing 102617, China*

*^3^ College of Materials Science and Engineering, Beijing University of Technology, Beijing, 100124, China*

*^4^ Institute of High Energy Physics, Chinese Academy of Sciences, Beijing 100049, China*

Table S1. XAFS fitting parameters at the Zn K-edge for various samples (*Ѕ*_0_^2^=0.87 from Zn-foil).

| Specimen | Shell | N | *d*(Å) | σ^2^(Å^2^) | R-factor |
| --- | --- | --- | --- | --- | --- |
| A-ZnO | Zn-O | 3.9±0.49 | 1.96±0.02 | 0.0076 | 0.0278 |
|  | Zn-Zn | 11.8±0.35 | 3.22±0.02 | 0.0080 |  |
| 400 ℃ | Zn-O | 3.5±0.37 | 1.96±0.01 | 0.0048 | 0.0284 |
|  | Zn-Zn | 11.7±0.36 | 3.22±0.02 | 0.0079 |  |
| 600 ℃ | Zn-O | 3.5±0.36 | 1.96±0.02 | 0.0045 | 0.0272 |
|  | Zn-Zn | 11.6±0.33 | 3.23±0.02 | 0.0060 |  |

*N*: coordination number; *d*: bond length; *σ*^2^: the mean square disorder at room temperature; R-factor: goodness of fit. Error bounds that characterize the structural parameters obtained by XAFS spectroscopy were estimated as N±20%; R±1%; σ^2^±20%.


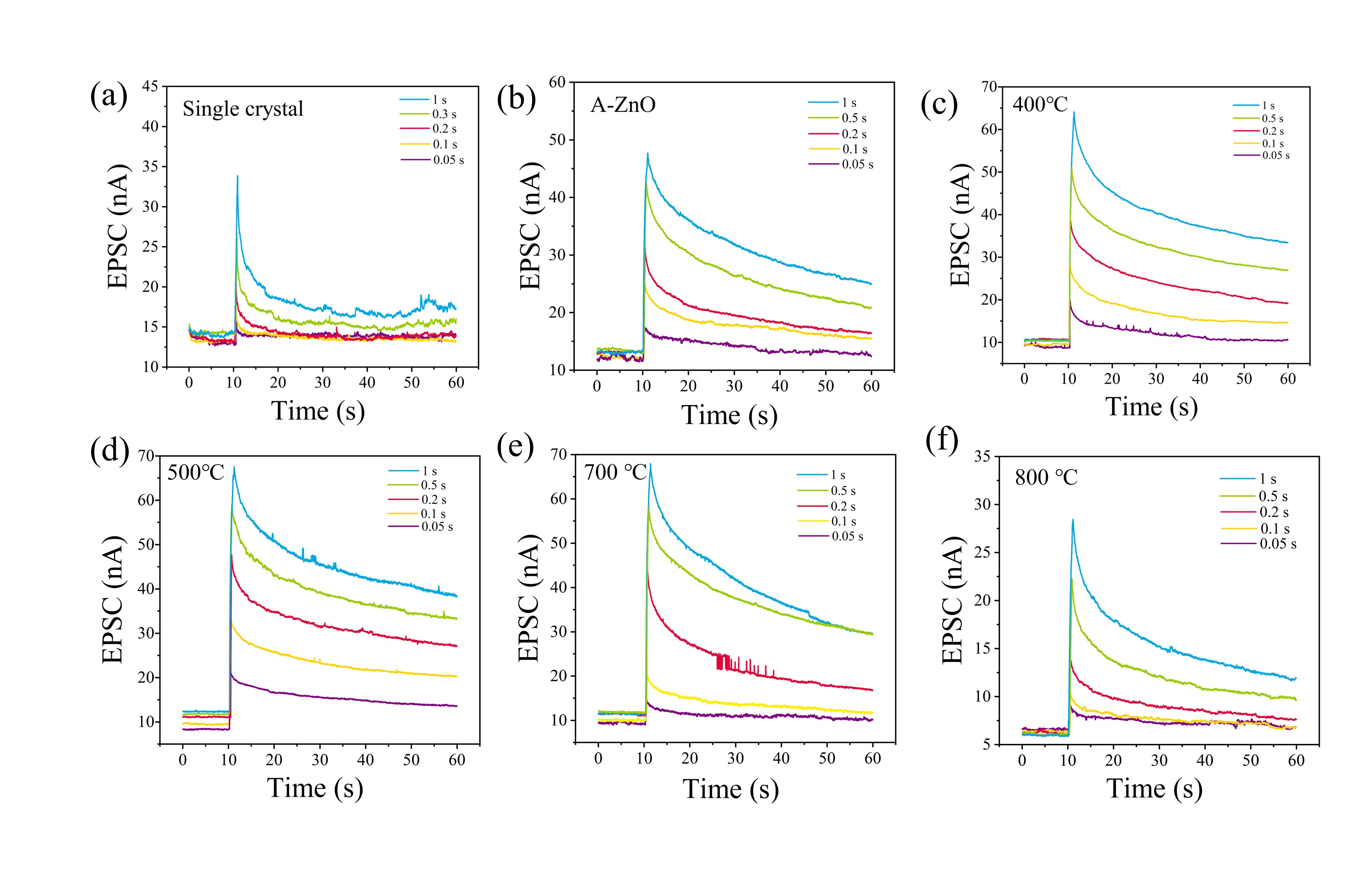


Fig. S1. Dependence of the light pulse duration time on the EPSC of the as-grown and annealed A-ZnO microtubes: (a) ZnO bulk single crystal, (b) as-grown, (c) 400 °C annealed, (d) 500 °C annealed, (e) 700 °C annealed, and (f) 800 °C annealed, respectively. A light pulse of 355 nm with varied duration time was applied to the as-grown and annealed A-ZnO microtubes, wherein all pulses with an energy density of 0.45 mJ/cm^2^, an interval time of 0.05 s, and a read voltage of 10 mV.





Fig. S2. Dependence of various annealing temperature on the EPSC response. A light pulse of 355 nm with a duration time of 1 s, an energy density of 0.45 mJ/cm^2^, an interval time of 0.05 s, and a read voltage of 10 mV.

Table S2. The light pulse duration time dependence of relative EPSC. The unit of EPSC is nA.

| Specimen | 0.05 | 0.1 | 0.2 | 0.5 | 1 |
| --- | --- | --- | --- | --- | --- |
| Bulk | 3.7 | 5.2 | 8.4 | 12.5 | 20.9 |
| A-ZnO | 6.2 | 13.8 | 18.3 | 29.5 | 35.8 |
| 400 ℃ | 10.1 | 19.9 | 28.8 | 41.2 | 54.5 |
| 500 ℃ | 11.4 | 25.4 | 36.7 | 47.6 | 56.8 |
| 600 ℃ | 55.9 | 77.8 | 95.9 | 118.6 | 133.0 |
| 700 ℃ | 3.6 | 14.6 | 33.8 | 46.4 | 56.1 |
| 800 ℃ | 3.2 | 4.0 | 8.2 | 16.3 | 22.5 |

Table S3 The enhancement of EPSC, and the memory of EPSC after removing illumination.

| Light condition | Bulk | A-ZnO | 400 ℃ | 500 ℃ | 600 ℃ | 700 ℃ | 800 ℃ |
| --- | --- | --- | --- | --- | --- | --- | --- |
| Under light | 1.6 | 3.0 | 5.5 | 5.2 | 12.1 | 4.7 | 3.8 |
| Removing light | 0.5 | 1.1 | 2.3 | 2.5 | 6.0 | 1.5 | 1.0 |


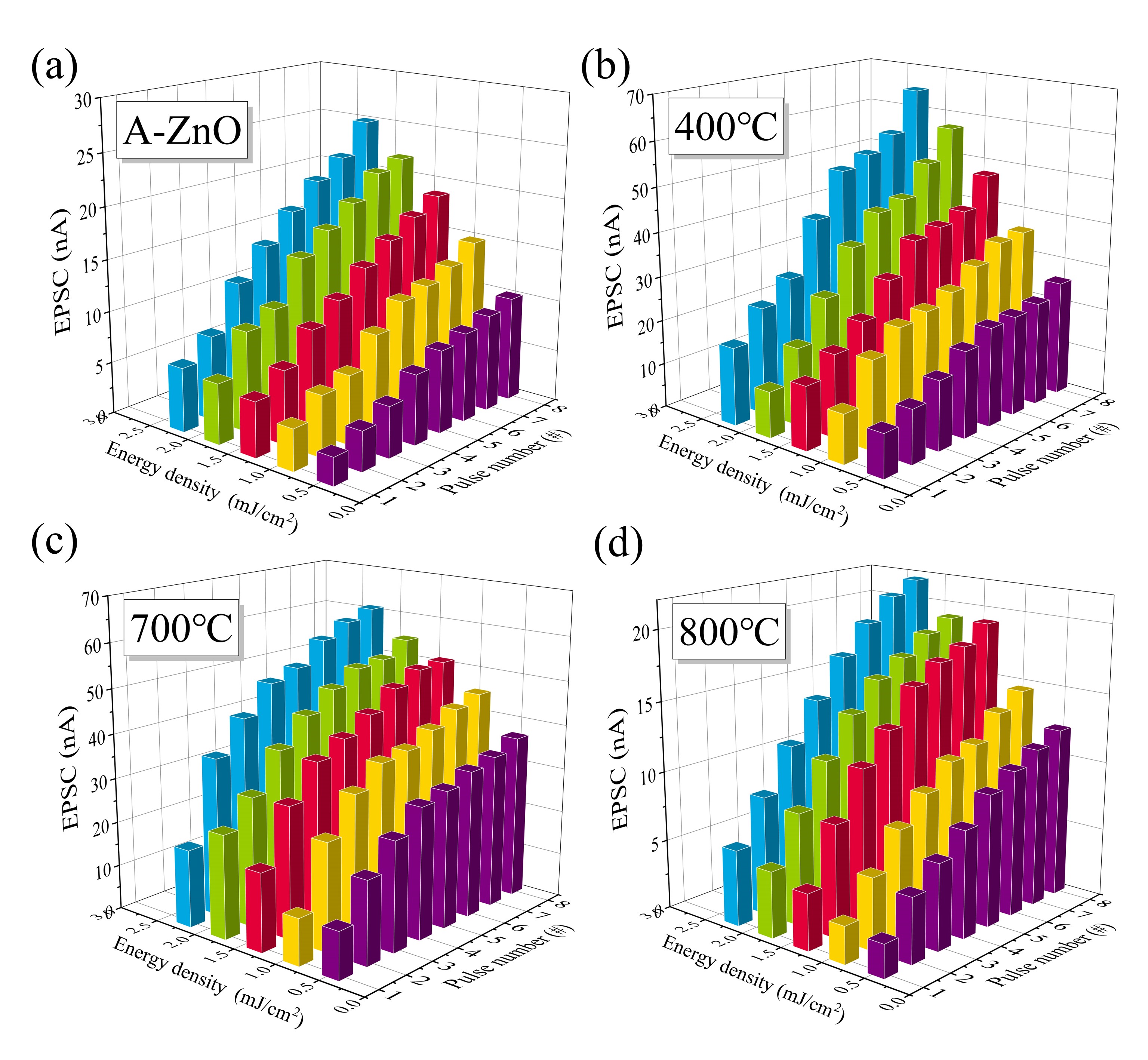


Fig. S3 The light pulse of number and energy density dependence of the as-grown and annealed A-ZnO microtubes: (a) as-grown, (b) 400 °C annealed, (c) 700 °C annealed, and (d) 800 °C annealed, respectively. Consecutive eight light pulses of 355 nm were applied to the as-grown and annealed A-ZnO microtubes, wherein all pulses with a duration time of 0.05 s, an interval time of 0.5 s, energy density ranging from 0.45 mJ/cm^2^ to 2.24 mJ/cm^2^, and a read voltage of 10 mV.





Fig. S4 Dependence of various annealing temperature on the EPSC response. A light pulse of 355 nm with a duration time of 0.05 s, an energy density of 2.24 mJ/cm^2^ an interval time of 0.5 s, and a read voltage of 10 mV.

Table S4. The enhancement EPSC fold of the as-grown and annealed A-ZnO microtubes, under the eighth light pulse energy density of 2.24 mJ/cm^2^.

| Light condition | As-grown | 400 ℃ | 600 ℃ | 700 ℃ | 800 ℃ |
| --- | --- | --- | --- | --- | --- |
| Under light | 0.9 | 5.5 | 17.6 | 4.5 | 2.1 |

The PPF behavior of the as-grown and annealed A-ZnO microtubes is stimulated by a pair of continuous light pulses with *Δt* ranging from 0.05 s to 10 s, an energy intensity of 0.45 mJ/cm^2^, a duration time of 0.05 s, and a read voltage of 10 mV, as shown in Fig.3(g) and Fig.S5-S8.


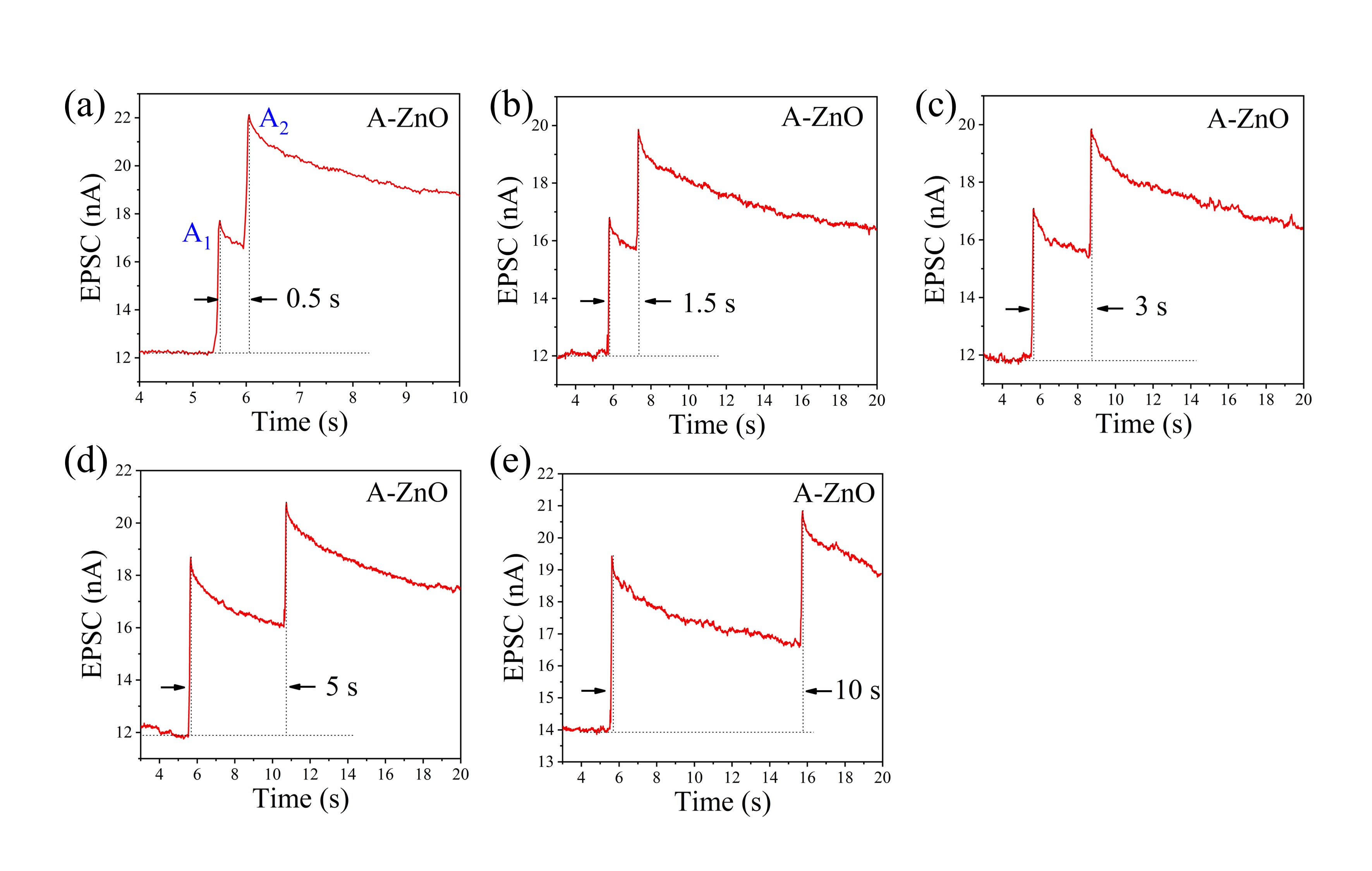


Fig. S5 The light pulse interval time dependence of the as-grown A-ZnO microtubes. Interval time of: (a) 0.5s, (b) 1.5s, (c) 3s, (d) 5s, and (e) 10s, respectively.


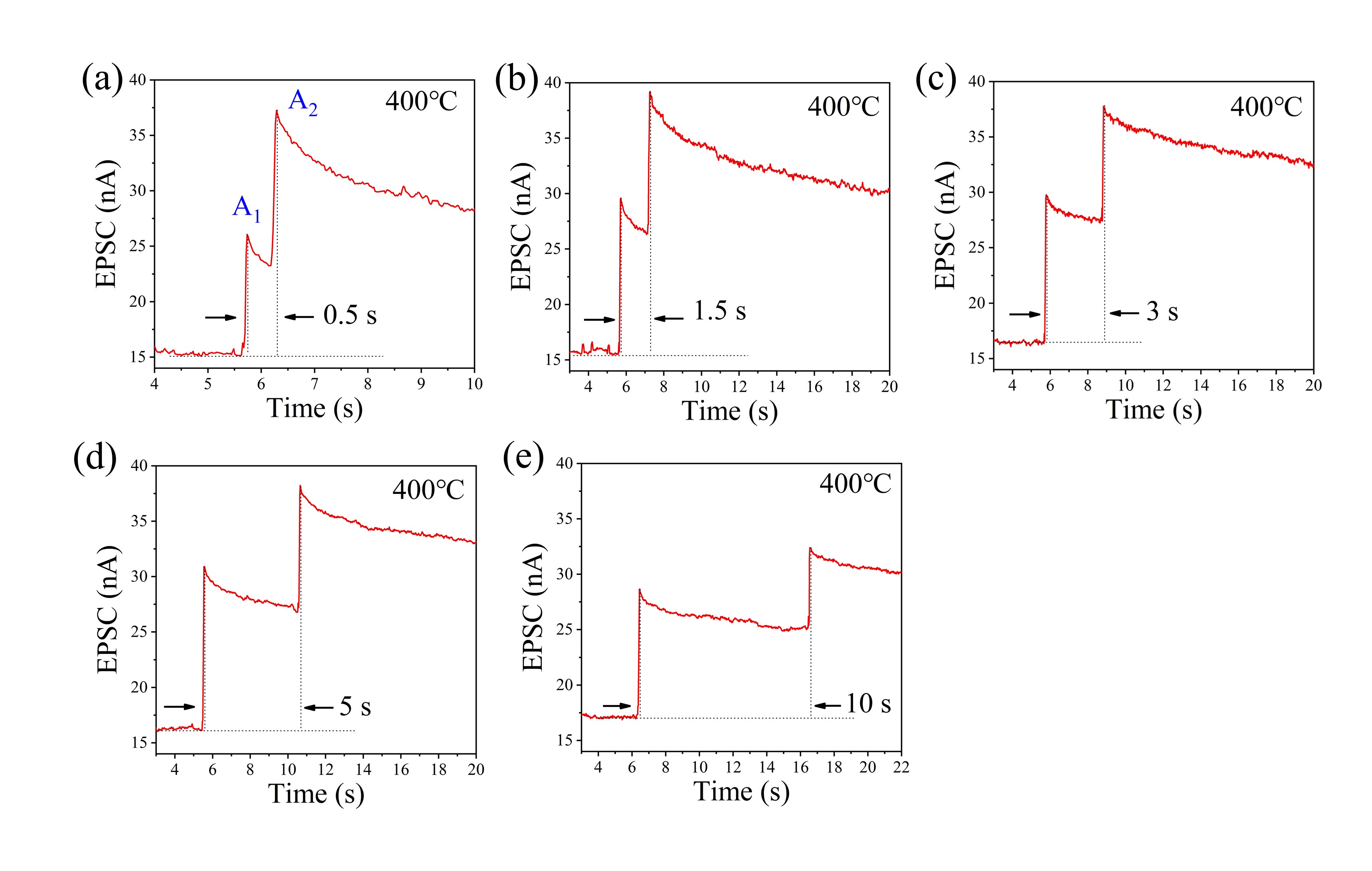


Fig. S6 The light pulse interval time dependence of the 400 °C annealed A-ZnO microtubes. Interval time of: (a) 0.5s, (b) 1.5s, (c) 3s, (d) 5s, and (e) 10s, respectively.


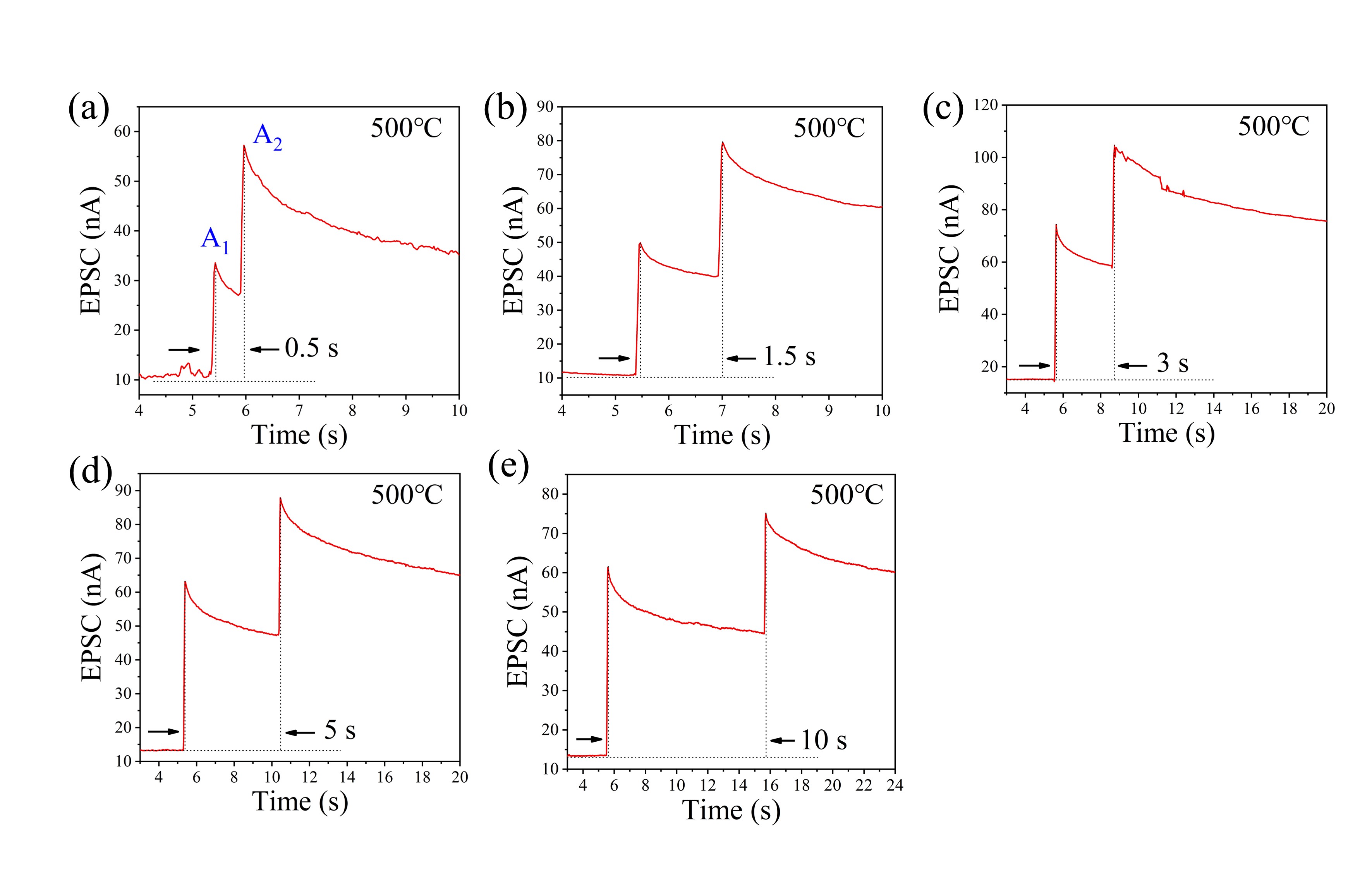


Fig. S7 The light pulse interval time dependence of the 500 °C annealed A-ZnO microtubes. Interval time of: (a) 0.5s, (b) 1.5s, (c) 3s, (d) 5s, and (e) 10s, respectively.


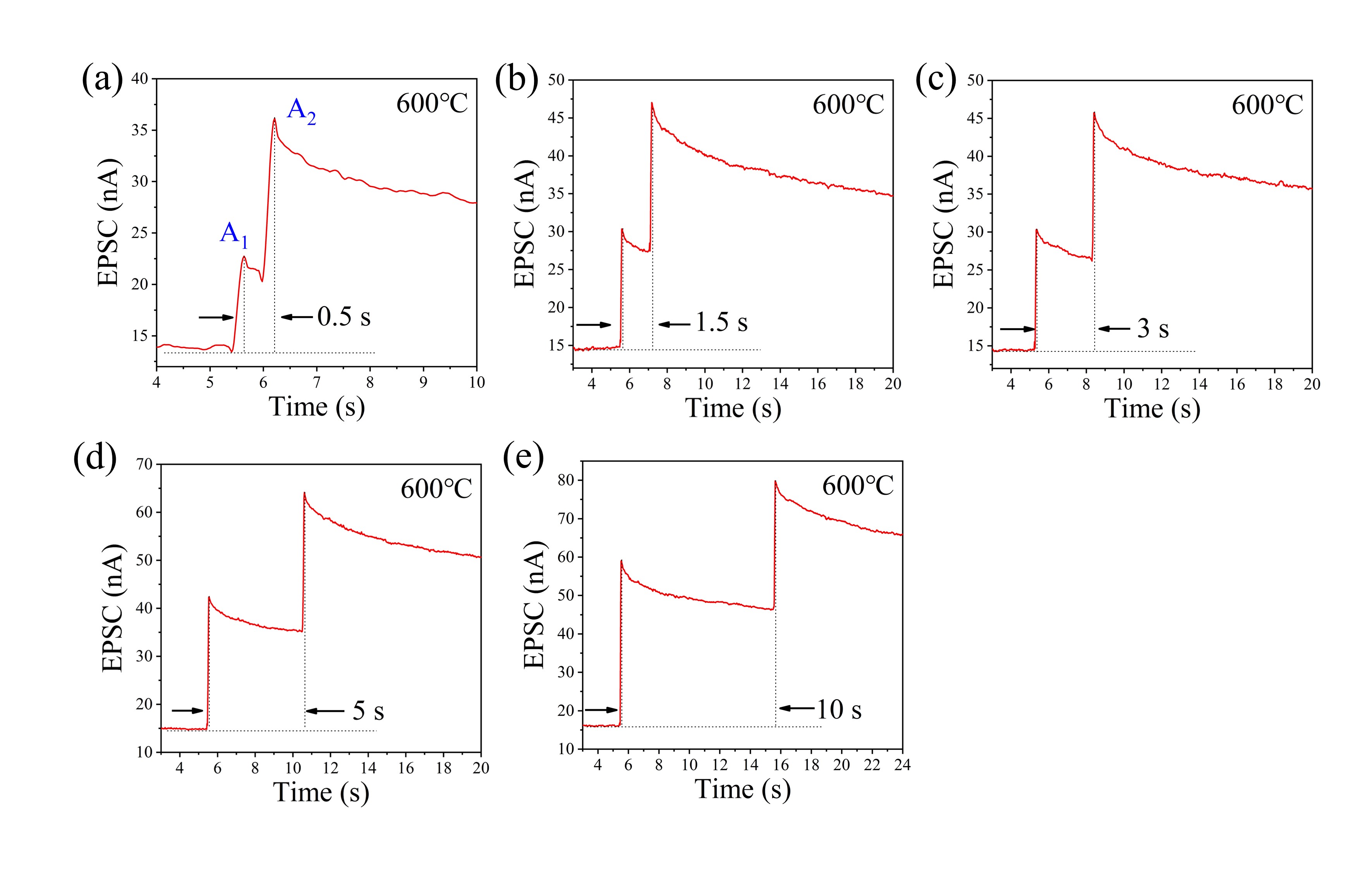


Fig. S8 The light pulse interval time dependence of the 600 °C annealed A-ZnO microtubes. Interval time of: (a) 0.5s, (b) 1.5s, (c) 3s, (d) 5s, and (e) 10s, respectively.

Table S5. PPF index various with the interval time.

| Specimen | 0.05 | 1.5 | 3 | 5 | 10 |
| --- | --- | --- | --- | --- | --- |
| A-ZnO | 180 | 164 | 152 | 131 | 126 |
| 400 ℃ | 207 | 170 | 160 | 150 | 132 |
| 500 ℃ | 203 | 176 | 150 | 150 | 128 |
| 600 ℃ | 238 | 206 | 198 | 178 | 148 |

Table S6. PPF index of interval time of 0.05 s.

| Specimen | τ_1_(s) | τ_2_(s) |
| --- | --- | --- |
| A-ZnO | 1.67 | 60.55 |
| 400 ℃ | 0.30 | 41.04 |
| 500 ℃ | 0.26 | 30.25 |
| 600 ℃ | 0.26 | 33.79 |

Table S7. Comparison of the PPF index of the 600 ℃ A-ZnO microtubes with literatures report.

| Device | PPF index | τ_1_ (s) | τ_2_ (s) | Ref. |
| --- | --- | --- | --- | --- |
| ZnO/Al_2_O_3_/CdS | 140 | 1.5 s | 27.9 s | [1] |
| Au/ZnO:N/IGZO/TiN | 115 | 5.1 s | 0.1 s | [2] |
| ITO/CuAlO_2_@ZnO/ITO | 160 | 1.4 s | 32.2 s | [3] |
| ZnO/HfO_x_ | 175 | - | - | [4] |
| ZnOnanosheet | 170 | - | - | [5] |
| 600 ℃ A-ZnO microtube | 238 | 0.26 | 33.79 | This work |


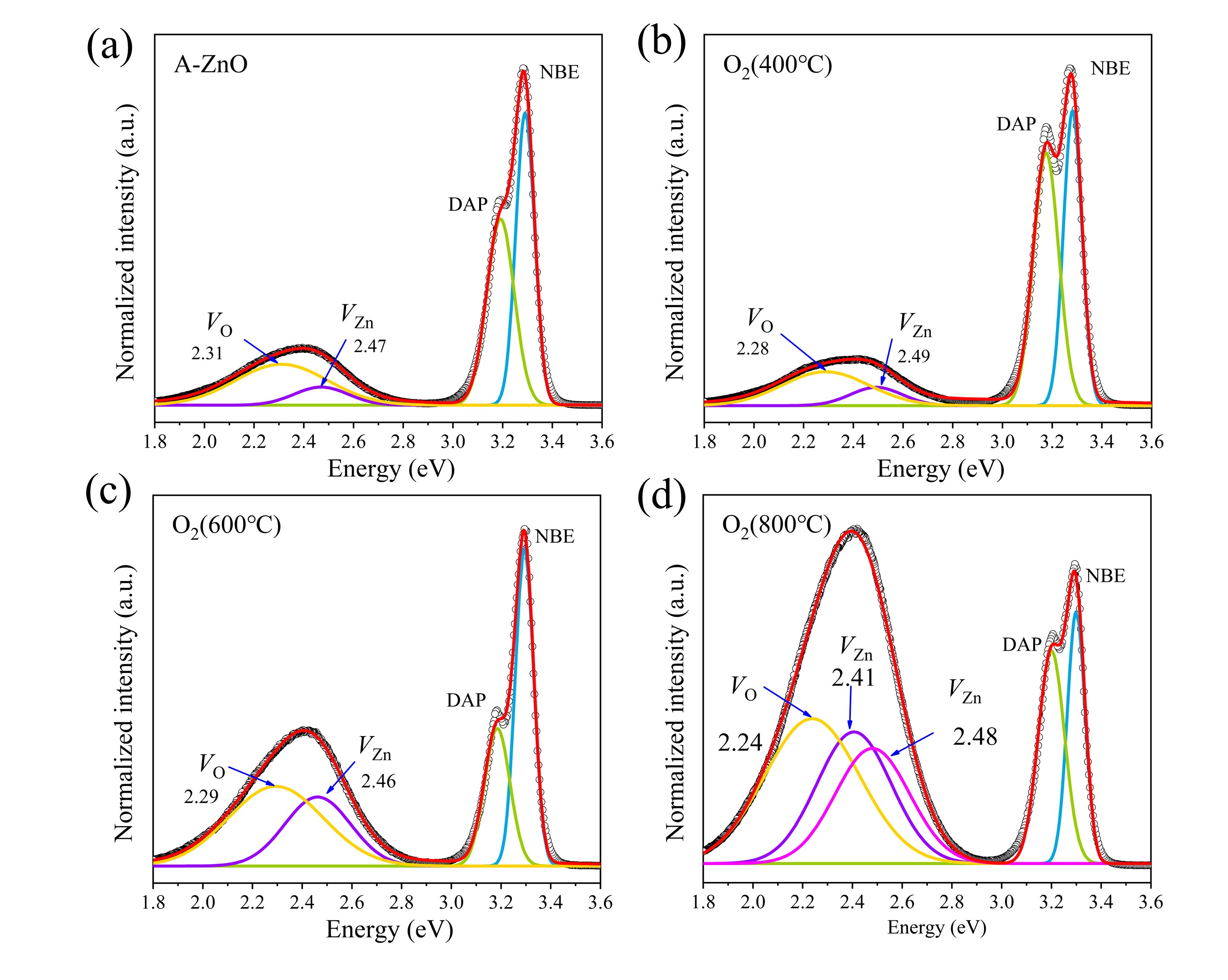


Fig. S9 PL spectra of the as-grown and annealed A-ZnO microtube at 300 K: (a) as-grown, (b) 400 °C annealed, (c) 600 °C annealed, and (d) 800 °C annealed, respectively.

Table S8. Summarized intensities ratios of the PL spectra of the as-grown and annealed A-ZnO microtubes by Gaussian fitting distributions.

| Specimen | *V*_O_ (eV) | I*_V_*_O_ */*I*_NBE_* | *V_Zn_* (eV) | I*_VZn_ /*I*_NBE_* |
| --- | --- | --- | --- | --- |
| A-ZnO | 2.31 | 0.12 | 2.47 | 0.05 |
| 400 ℃ | 2.28 | 0.10 | 2.49 | 0.06 |
| 600 ℃ | 2.29 | 0.24 | 2.46 | 0.21 |
| 800 ℃ | 2.24 | 0.44 | 2.41 | 0.40 |





Fig. S10 PL spectra measured in the temperature range of 80–300 K for A-ZnO microtubes annealing temperature of (a-c) 400 ℃ and (d-f) 600 ℃, respectively.

Table S9. Fitting results of PALS for the as-grown and 600 ℃ annealed A-ZnO microtubes.

| Specimen | *τ*_1_ (ps) | | *I*_1_(%) | *τ*_2_ (ps) | *I*_2_(%) | *τ*_3_ (ps) | *I*_3_(%) | *τ*_ave_ |
| --- | --- | --- | --- | --- | --- | --- | --- | --- |
| As-grown | 135.9 | 39.9 | | 247.3 | 59.7 | 1059 | 0.372 | 205.8 |
| 600 ℃ annealed | 129.7 | 32.7 | | 246.5 | 66.8 | 1003 | 0.498 | 212.1 |

Table S10. The binding energy of Zn 2*p* and O 1*s* from the as-grown and annealed A-ZnO microtube

| Specimen | *O_C_* | *O_V_* | *O_L_* | Zn *(2p_1/2_)* | Zn *(2p_3/2_)* |
| --- | --- | --- | --- | --- | --- |
| As-grown | 531.95 | 531.09 | 529.80 | 1044.15 | 1021.03 |
| 400 ℃ annealed | 532.95 | 531.69 | 530.10 | 1044.54 | 1021.42 |
| 600 ℃ annealed | 531.96 | 530.94 | 530.16 | 1044.63 | 1021.48 |


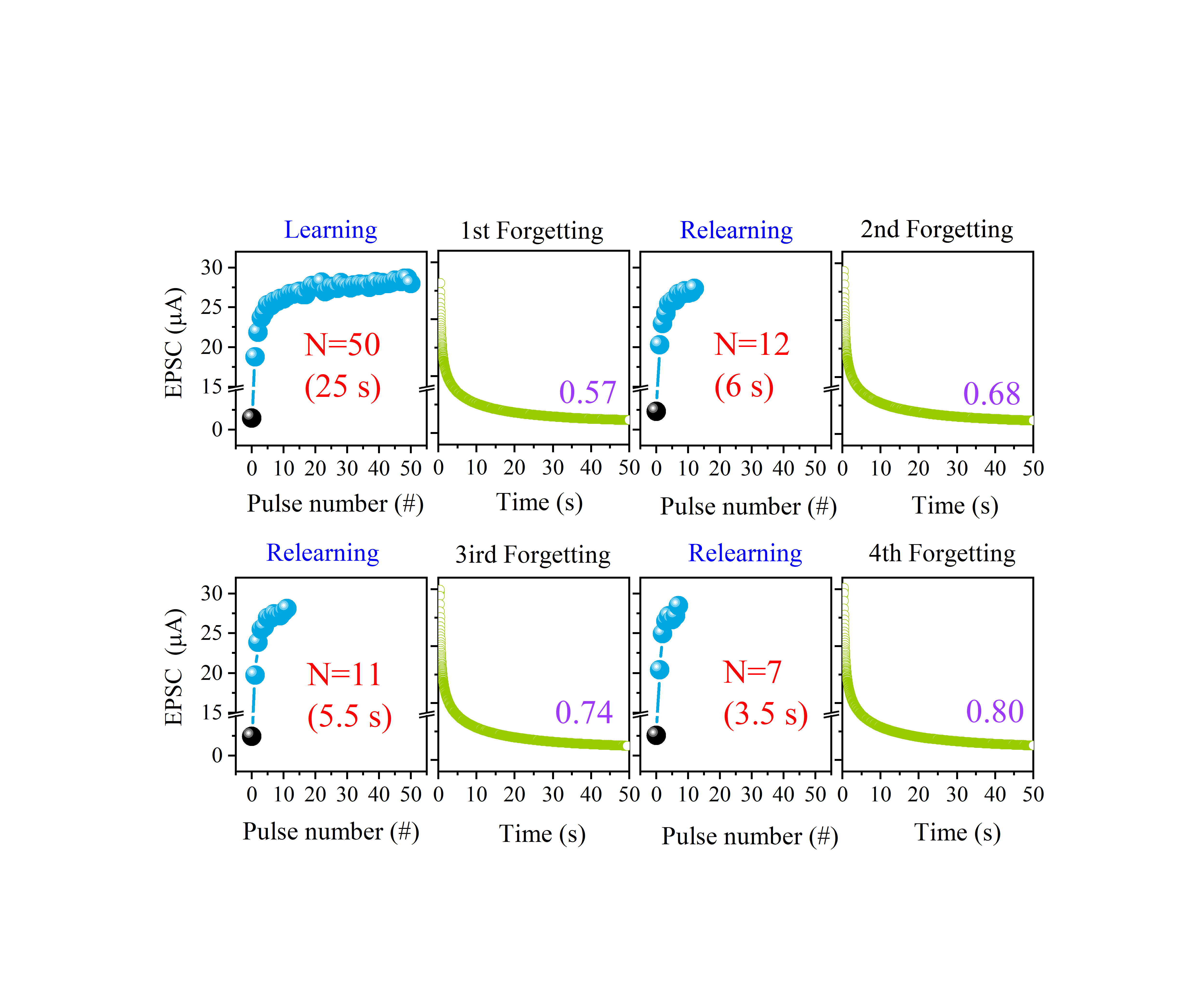


Fig. S11 Simulation of learning-forgetting-relearning-forgetting behavior of annealing 600 ℃ of A-ZnO microtube device (UV pulses of 355 nm with an energy intensity of 5.97 mJ/cm^2^, a duration time of 0.5 s, an interval time of 0.5 s, and a read voltage of 10 mV).

References

1. X. Han, Y. Zhang, ZH. Huo, X. Wang, G. Hu, ZH. Xu, H. Lu, Q. Lu, X. Sun, L. Qiu, P. Yan, C. Pan, A two-terminal optoelectronic synapses array based on the ZnO/Al_2_O_3_/CdS heterojunction with strain-modulated synaptic weight, Adv. Electron. Mater. 9 (2023) 2201068.
2. Y. Sun, Q. Li, X. Zhu, C. Liao, Y. Wang, ZH. Li, S. Liu, H. Xu, W. Wang, In-sensor reservoir computing based on optoelectronic synapse. Adv. Intell. Syst. 5 (2023) 2200196.
3. CH. Li, N. Ilyas, J. Wang, Y. Li, H. Luo, D. Li, D. Gu, F. Liu, Y. Jiang, W. Li, Nanostructured CuAlO_2_@ZnO optoelectronic device for artificial synaptic applications. Appl. Surf. Sci. 611 (2023) 155682.
4. X. Shan, CH. Zhao, Y. Lin, J. Liu, X. Zhang, Y. Tao, CH. Wang, X. Zhao, ZH. Wang, H. Xu, Y. Liu, Optoelectronic synaptic device based on ZnO/HfO_x_ heterojunction for high-performance neuromorphic vision system. Appl. Phys. Lett. 121 (2022) 263501.
5. V. Krishnamurthi, T. Ahmed, M. Mohiuddin, A. Zavabeti, N. Pillai, C. Mcconville, N. Mahmood, S. Walia, A visible-blind photodetector and artificial optoelectronic synapse using liquid-metal exfoliated ZnO nanosheets. Adv. Optical. Mater. 9 (2021) 2100449.
